# DFT Modelling of Molecular Structure, Vibrational and UV-Vis Absorption Spectra of T-2 Toxin and 3-Deacetylcalonectrin

**DOI:** 10.3390/ma15020649

**Published:** 2022-01-15

**Authors:** Dmitrii Pankin, Mikhail Smirnov, Anastasia Povolotckaia, Alexey Povolotskiy, Evgenii Borisov, Maksim Moskovskiy, Anatoly Gulyaev, Stanislav Gerasimenko, Aleksandr Aksenov, Maksim Litvinov, Alexey Dorochov

**Affiliations:** 1 Center for Optical and Laser Materials Research, St. Petersburg State University, Ulianovskaya 5, 198504 St. Petersburg, Russia; dmitrii.pankin@spbu.ru (D.P.); eugene.borisov@spbu.ru (E.B.); 2 Solid State Physics Department, Physical Faculty, St. Petersburg State University, Ulianovskaya 1, 198504 St. Petersburg, Russia; m.smirnov@spbu.ru; 3 Institute of Chemistry, St. Petersburg State University, Universitetskii pr. 26, 198504 St. Petersburg, Russia; alexey.povolotskiy@spbu.ru; 4 Federal Scientific Agro-Engineering Center VIM, 1st Institutskiy proezd 5, 109428 Moscow, Russia; max-moskovsky@yandex.ru (M.M.); tomasss1086@mail.ru (A.G.); stanislav.mkm@gmail.com (S.G.); 1053vim@mail.ru (A.A.); litvvinov.max@yandex.ru (M.L.); dorokhov@rgau-msha.ru (A.D.)

**Keywords:** Mid IR absorbance, T-2, 3-deacetylcalonectrin, toxin, vibrational modes, UV-Vis absorbance, DFT calculations

## Abstract

This paper discusses the applicability of optical and vibrational spectroscopies for the identification and characterization of the T-2 mycotoxin. Vibrational states and electronic structure of the T-2 toxin molecules are simulated using a density-functional quantum-mechanical approach. A numerical experiment aimed at comparing the predicted structural, vibrational and electronic properties of the T-2 toxin with analogous characteristics of the structurally similar 3-deacetylcalonectrin is performed, and the characteristic spectral features that can be used as fingerprints of the T-2 toxin are determined. It is shown that theoretical studies of the structure and spectroscopic features of trichothecene molecules facilitate the development of methods for the detection and characterization of the metabolites.

## 1. Introduction

The T-2 toxin and 3-deacetylcalonectrin are A-type trichothecene micotoxines [1,2,3]. These toxins are produced by various *Fusarium* fungi [1,2]. In particular, 3-deacetylcalonectrin has been extracted from *F. graminearum* and *F. culmorum* [4], while the T-2 toxin has been excreted by *F. sporotrichioides*, *F. tricinctum* and *F. poae* [5]. The T-2 is the most toxic of the trichothecenes and poses a serious danger to humans and animals by ingestion of contaminated grains and their processed products [5]. Unlike most biological toxins, T-2 mycotoxin can be absorbed through intact skin. The toxin-releasing moulds grow on a large variety of cereals. Certain toxins produced by the genus *Fusarium* fungi are characteristic of certain types of cereals, for example, the T-2 toxin often appears in oats [6]. Trichothecenes can cause severe skin disease or intestinal mucosa irritation and diarrhoea. The suppression of animal immune systems has also been noted as a chronic effect of T-2 toxin contamination.

The production of the T-2 mycotoxin occurs during plant infection by the fungus genus *Fusarium*, followed by deep toxin penetration into the grain [7]. The particular importance in T-2 mycotoxin identification is associated with extensive food production from grains that are potentially susceptible to *Fusarium* spp. infection, such as barley, wheat and oat [2].

Theoretical studies of the spectroscopic features of the trichothecene structure could facilitate characterization and methods of detection development. To date, the identification and investigation of the T-2 toxin structure, including immunochemical and chromatography (gas chromatography, thin-layer chromatography, high-performance liquid chromatography) techniques [2,8,9] and different nuclear magnetic resonance methods [2,7,10] and X-ray diffraction spectroscopy [11], has been carried out.

Optical methods can also provide valuable information on the mycotoxin structure and may be very useful for the identification of these metabolites. Currently, UV-Vis absorption and fluorescence of toxin derivatives [9], coupled with chromatographic methods, are used for toxin identification. Furthermore, it has been demonstrated that a complex approach that combines GC and FTIR (GC-FTIR) techniques is rather promising for detection of the auxiliary molecular fragments containing hydroxyl, acetate and carbonyl groups [3].

In the last decade, several articles have demonstrated the availability of the liquid chromatography and Raman [12], thin-layer chromatography (TLC) and SERS [13] and HPLC & SERS combinations [14]. At the same time, only a few attempts have been made at relatively low T-2 toxin concentration measurements by vibrational techniques; see, e.g., [15].

The vibrational spectroscopy technique, i.e., IR absorption and Raman scattering (including SERS), is very sensitive to the structural peculiarities of the toxins [16,17]. Application of the SERS technique allows one to significantly reduce the detectable concentration [18,19,20,21,22]. However, the efficiency of the vibrational spectroscopy approach strongly depends on the availability of information on characteristic spectral features which can be used as fingerprints of the compound under study. At present, the most reliable interpretation of the vibrational spectra can be obtained from first-principle quantum mechanical calculations. Moreover, such calculations may provide valuable information on the electronic structure, which can be useful for interpreting the optic (UV-Vis absorption and fluorescence) spectra of toxin derivatives. All these circumstances necessitate further and deeper investigations of the electronic structure and vibrational spectra of the T-2 toxin, which is vital from a practical point of view.

This study is aimed at modelling the electronic and vibrational spectra of the T-2 toxin. Such theoretical results can serve as guidance for further experimental studies. To verify the reliability of the chosen computational method, we first applied it to study the structural, electronic and vibrational properties of the closely related metabolite 3-deacetylcalonectrin, which has already been studied by the FTIR absorbance technique [3]. Ensuring that the calculations and experiment are in good agreement, we turn to modelling and a detailed interpretation of the IR and UV spectra of the T-2 toxin. The main aim of the study was a determination of characteristic spectral features which can be used as the fingerprints of this compound.

## 2. Methodological Part

### Calculation Details

The DFT calculations were performed with the aid of Gaussian G09W Rev. C software (Gaussian Inc, Wallingford, CT, USA) [23]. The gas-phase molecule geometry optimization was performed with B3LYP exchange-correlation functional, which has been tested previously in trichothecenes electronic properties studies [24,25]. The 6-311G(2d,p) basis set, with ultrafine integration grid, was used for the calculations [24,25]. Molecular geometry was optimized with the convergence criteria of maximal and RMS forces and displacements. The stability was tested by the absence of imaginary vibrational frequencies. For better agreement between calculated and experimental data, the calculated vibrational frequencies were multiplied by a scaling factor of 0.983 for both T-2 and 3-deacetylcalonectrin. The IR peak half-width at half-height for the demonstrated spectra was 4 cm^−1^. For convenient comparison with the experiment in this paper, the scaled frequencies are discussed.

The excitation energies and oscillator strength for 60 singlet excited states were simulated with the use of the TD-DFT approach [26]. The UV-Visible absorption spectra were simulated by assuming a Gaussian band shape with a peak width of 0.3 eV. The Polarizable Continuum Model (PCM) [27] was used for simulating the solvent effect in the methanol media.

All calculations were carried out for isolated molecules, whereas the computational results were compared with experimental data on the structure and IR absorption of crystalline materials. Such inconsistency is due to two circumstances. First, both of the studied compounds are rather complicated. There are 536 atoms in the unit cell of the T-2 toxin crystal. Hence, rigorous periodic DFT modelling is hardly realistic. Second, the intermolecular binding in the T-2 toxin crystal is mainly governed by weak van-der Waals forces [11]. Hence, periodic DFT modelling is unlikely to provide any fundamentally new information on the spectra of the internal vibrational modes. Our main goal is to determine characteristic lines in the IR absorption spectra. In relation to this, internal vibrational modes are mostly related to the characteristic lines in the middle-frequency spectral region. Previous molecular crystal studies have evidenced that the frequencies of the intra-molecular vibrational modes weakly depend on the crystalline environment in the absence of strong inter-molecular interaction. This assumption is widely used in discussing the vibrational states of the molecular crystals [28,29,30,31].

## 3. Results

### 3.1. Structure

#### 3.1.1. Structural Peculiarities of Fused Rings in Free Molecules

**Figure 1:**
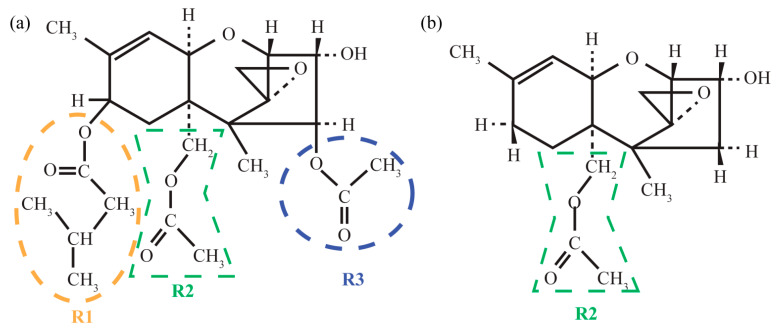
Schematic representation of T-2 (**a**) and 3-deacetylcalonectrin (**b**) toxin molecules. Orange, green and blue contours highlight the R1, R2 and R3 groups.

The molecular structures of T-2 and 3-deacetylcalonectrin are schematically represented in Figure 1. One can see that the core structures of both molecules consist of three fused rings: cyclohexene (A-ring), tetrahydropyran (B-ring) and cyclopentane (C-ring). The biological activity of the toxins is related to characteristic structural features, which are the epoxy ring, the hydroxyl groups and the acetate group (R2) [24]. The T-2 toxin molecule is bigger—it contains two additional functional groups, R1 and R3. This leads to additional conformational diversity as compared to 3-deacetylcalonectrin [7].

The crystal structure of the T-2 toxin was investigated by the single crystal XRD method [11]. It was found that the lattice is orthorhombic and corresponds to the P2_1_2_1_2_1_ space group, with eight molecules in the unit cell. The asymmetric unit contains two molecules (assigned as molecule I and molecule II). They differ in the dihedral angles of the 3-methylbutanoate group (R1). At the same time, it was noted that the molecular structure strongly depends on the environment and on the state of matter [7].

In view of the conformational diversity, we have decided to look for the lowest energy configuration for both molecules. Geometry optimization was carried out for isolated molecules. This corresponds to non-interacting molecule approximation. Such an approach allows us to compare the structural characteristics of the T-2 toxin and the closely related 3-deacetylcalonectrin molecules. Moreover, it is possible to discuss differences in the structure and vibrational spectra of these molecules.

**Figure 2:**
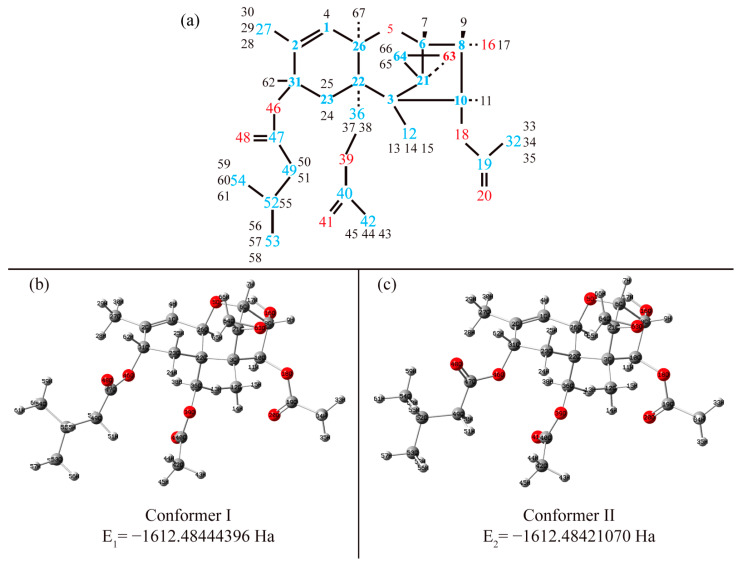
Atomic labels for T-2 molecule (blue, red and black numbers are representation of carbon, oxygen and hydrogen atoms, respectively) (**a**) and stereochemical pictures for conformer I (**b**) and conformer II (**c**).

The XRD data for the T-2 crystals indicate the existence of two different molecular configurations. We performed geometry optimization starting from both of them and obtained two stable configurations. One (conformer I) corresponds to the lowest energy structure and is shown in Figure 2b. The other (conformer II) differs in the orientation of the functional groups and is shown in Figure 2c.

One can see that the core structures (i.e., the structure of the fused rings) of all of the molecules are similar. The T-2 conformers are similar in the following: if a plane is conventionally drawn along the A-ring, the dipole moment of the C=O bond in 3-methylbutanoate is directed downward, while in both acetate groups this dipole moment is directed upward in both optimized geometries. The most prominent difference between two conformers of T2 concerns the orientation of the 3-methylbutanoate group (R1). This is in line with experimental data [11] for two molecules in the asymmetric unit. The result allows us to suggest that the two conformers found in our calculations correspond to two nonequivalent molecules in the crystal structure of T-2. The dihedral angles C23-C31-O46-C47, C22-C36-O39-C40 and C8-C10-O18-C19 for conformer I are equal to 87.3°, 172.7° and −143.5°, and for conformer II are equal to 119.1°, 161.8° and −142.4°, respectively. The above-mentioned angles for conformer I are close to those experimentally observed in the XRD study [11]; for molecule I, the angles are equal to 83.4°, 169.9° and −138.8° respectively. However, the experimental values for the second molecule are equal to 118.0°, 169.0° and −116.7°. The divergence in the C8-C10-O18-C19 angle for conformer II is clearly manifested (see Table S1). The observed divergence can be explained by intermolecular interaction, which is not taken into account when performing calculations in the gas phase. Based on NMR studies, conformational flexibility in the 3-methylbutanoate and acetate was noted [7]. This structural dissimilarity moderately affects the dipole moment. The total dipole moment of conformer I is equal to 2.257 Debye, and it equals 2.3315 Debye for conformer II, which is approximately 3.3% higher. The difference in total energy is approximately 0.146 kcal/mol. This is relatively small comparing to the thermal energy at 300 K, which is equal to approximately 0.6 kcal/mol. Such a low energy difference makes the thermodynamic probabilities for the two conformers almost equal.

The core structures (i.e., the three fused rings) in the 3-deacetylcalonectrin and T-2 toxin molecules have several common structural features.

According to Table S1, the shortest bond in the cyclohexene ring (A-ring), corresponding as expected to the C1=C2 double bond, is approximately equal to 1.330 Ǻ for both T-2 toxin and 3-deacetylcalonectrin. The longest bond among the single carbon bonds is predicted at the border of the tetrahydropyran type ring (B-ring); it equals 1.555 Ǻ for the T-2 toxin (C22-C26) and 3-deacetylcalonectrin (C23-C19). According to XRD, the longest C–C bonds are C22-C26 and C22-C23 for the T-2 toxin and C19-C20 and C19-C23 for 3-deacetylcalonectrin. The lengths of the remaining C–C bonds take intermediate values between 1.501 and 1.555 Ǻ. The corresponding planar angles in both molecules have similar values. The large values of the plane angles refer to the C–C=C and C=C–C parts of the A-ring, which is explained by the relatively higher electron density localized in the double carbon bond compared to the single bond. The smallest flat angle is approximately 108° and is formed by two carbon atoms adjoining the B-ring and carbon in the CH_2_ group (C23-C22-C26).

According to theory, the greatest relative differences between the T-2 toxin molecule and the 3-deacetylcalonectrin occur in the dihedral angles with the carbon atom to which the 3-methylbutanoate group is attached. In the T-2 molecule the electrostatic repulsion between the R1 and R2 groups leads to a shift of the C31 atom. This changes the dihedral angle in the A-ring from almost zero (C23-C1=C2-C28 angle in 3-deacetylcalonectrin) to approximately 1.6–1.7° (C26-C1-C2-C31 angle in T-2 toxin conformers). According to calculations, the largest difference in dihedral angles takes place for the C2-C31-C23-C22 angle in both conformers of the T-2 toxin (about −38.7°) and the corresponding C2-C28-C20-C19 angle in 3-deacetylcalonectrin (approximately −46.1°); see Table S1.

In the XRD experiment, the values of the C2-C31-C23-C22 angle were −41.2° and −36.7° for the two molecules in the asymmetric unit [11]. The calculated values of the angle for both T-2 conformers is approximately −38.7^o^, which is closer to the averaged value over two experimental molecules.

The difference between the optimized bond lengths in the B-rings of the two toxin divergences is within a few thousandths of an angstrom. It is less than 0.62° for the planar angles.

**Figure 3:**
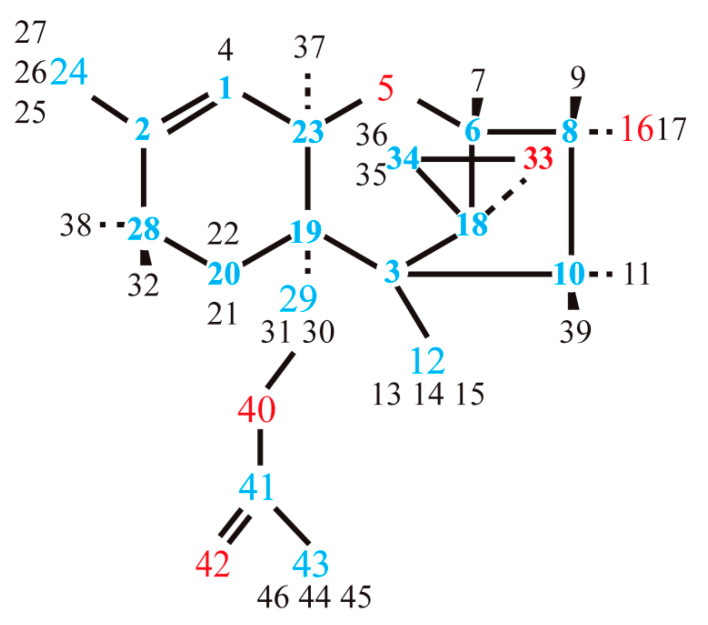
Atomic labels for the 3-deacetylcalonectrin molecule (blue, red and black numbers are representation of carbon, oxygen and hydrogen atoms, respectively).

Let us consider dihedral angles involving the C19 atom (in the notations of Figure 2a) and the C22 atom (in the notations of Figure 3). These angles have similar values (within 0.3° divergence) for the two T-2 toxin conformers. At the same time, the divergence increases up to 1–1.5° when comparing the angles of the T-2 toxin with the corresponding angles of 3-deacetylcalonectrin.

Such a small difference could be due to the R2 group attached to these carbon atoms. The electrostatic interaction of this group with the R1 and R3 groups nearby is small. The shortest bonds correspond to C–O in the B-ring. The C–C bonds in both types of toxins lie in the range of 1.51 to 1.59 Ǻ.

A slight overestimation in the calculated bond lengths in comparison with the X-ray structural data can be noted for the B-ring of the T-2 toxin. The largest error is approximately 1.7% for the C21-C6 bond. The highest divergences (up to 2°) were noted for the planar angles. The highest divergence for dihedral angles takes place for the C21-C6-O5-C26 angle.

According to theory, the values of bond lengths and planar and dihedral angles in the cyclopentyl ring (C-ring) are close in both T-2 conformers. Difference within 0.66% were manifested between the C–C bonds of the T-2 conformers and 3-deacetylcalonectrin. The largest value takes place for the C3-C10 bonds. The smallest difference between dihedral angles in the C-ring of the T-2 conformers and 3-deacetylcalonectrin takes place for the angle with the C10 atom (approximately 0.05°). For the remaining dihedral angles, the difference varies from 2.2° to 4.2°; see Table S1. The divergence between the theoretical and experimental values of the dihedral angles in the C-ring lies within 1–1.5° for the T-2 toxin molecule. The largest variance (approximately 2%) in bond lengths is noted for the C8-C10 bond.

The calculated C–O bond in the epoxy ring (E-ring) is shorter than in the experiment. We suppose that this is due to the absence of the hydrogen bond through the O63 atom. A significantly short contact between the OH group of molecule 1 and the oxygen atoms of the epoxy group of molecule 2 was noted in the real crystal structure [7].

#### 3.1.2. Arrangement of 3-Methylbutanoate and Acetate Groups

As stated above, the 3-methylbutanoate group (R1 group) has conformational flexibility. The calculated values of the dihedral angles near the A-ring are in good agreement with the experimental data. Additionally, the value of the C23-C31-C46-C47 angle fits the experiment for two conformers relatively well (the divergence lies within 4°). At the same time, the hydrogen bonds with the terminal CH_3_ group, which affects the dihedral angles C46-C47-C49-C52, were not taken into account in the gas phase calculations. In particular, these dihedral angles are equal to approximately −154° for both conformers, while the experimental data provide value of −77.7° for molecule 1 and −87.2° for molecule 2. At the same time, the difference between the experiment and theory for the dihedral angle C47-C49-C52-C53 lies within 8–10°. Presumably, the effect of this divergence involves the frequencies of stretching and bending vibrations in CH_3_, slightly increasing them due to the absence of hydrogen interaction. At the same time, the characteristic vibrational frequencies in the torsion angles with carbon atoms have relatively low values (usually less than 300 cm^−1^) and low polarity, which is expected to have a low influence on IR active modes.

For the R2 functional group, the highest divergence between theory and experiment concerns the value of the C22-C36-O39-C40 angle in conformers II of the T-2 toxin. The calculated value of this angle is equal to 172.7° for conformer I, whereas the experiment gives 169.9° for molecule 1. In turn, the calculated value of this angle is equal to 161.8° for conformer II, whereas the experiment gives 169.0° for molecule 2. The difference in dihedral angles C36-C39-C40-C42 is approximately 4.5°, while in C3-C22-C36-C39 it does not exceed 1.7°.

In the case of 3-deacetylcalonectrin, a similar functional group has flatter dihedral angles C22-C36-O39-C40 and C36-O39-C40-C42, which are close to 180^o^. The C3-C22-C36-O39 angle is less by 7–8°, which means a reversal of this radical towards the A-ring rather than in the direction of the methyl group attached to the B-ring.

The calculated parameters of the acetate group attached to the C-ring are very similar for both T-2 toxin conformers. The main differences concern the CO bond length, linking the acetate group and the C-ring, as well as the dihedral angle C8-C10-O18-C19, which determines the angle of rotation relative to the ring plane. For the T-2 toxin conformer I, the dihedral angle C8-C10-O18-C19 is equal to −143.5°, and the length of the C10-C18 bond is equal to 1.442°. This is close to experimental XRD data from which the corresponding dihedral angle is −138.8° and the bond length is 1.439 Ǻ.

Considering conformer II, the calculated dihedral angle C8-C10-O18-C19 and the length of the C10-C18 bond are equal to −142.4° and 1.442 Ǻ, which are close to conformer I. At the same time, the XRD data for molecule 2 provide a bond length of 1.46 Ǻ and a dihedral angle of −116.7°, which differ markedly from the analogue dihedral angle in conformer II.

Based on the aforementioned reasons, conformer I of the T-2 toxin, which is structurally close to one of the molecules obtained in XRD [3] and slightly more stable than conformer II, was chosen to model the IR absorption spectrum.

### 3.2. Vibrational Properties

Currently, experimental information on the vibrational properties of the T-2 toxin is rather scarce. Inversely, the vibrational states of the structural analogue of 3-deacetylcalonectrin have been thoroughly studied [3]. Thus, we have decided to perform a comparative analysis of the simulated vibrational spectra of these compounds, taking into account the experimental data of [3].

#### 3.2.1. 3-Deacetylcalonectrin Molecule

**Figure 4:**
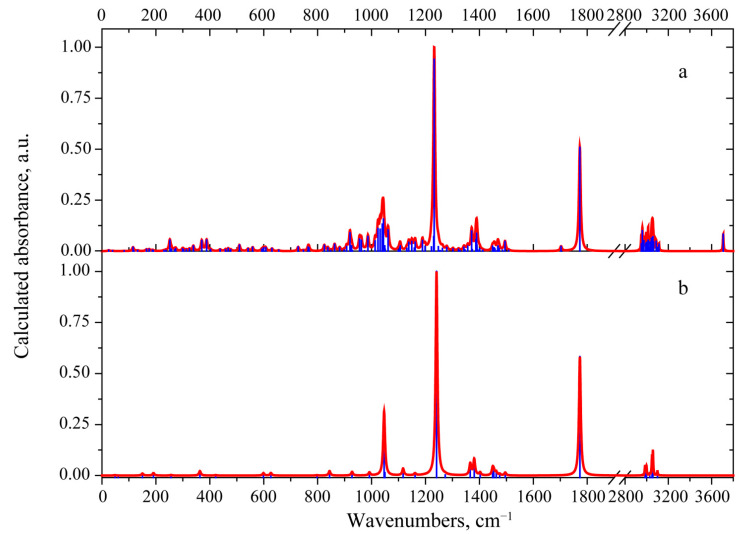
The calculated scaled IR absorbance spectrum of 3-deacetylcalonectrin (**a**) and ethyl acetate model (**b**).

The experimental IR absorption spectrum in the range 750–4000 cm^−1^ was reported in [3]. The spectral curve contains several absorption bands. Their frequency positions are listed in Table 1. Some of these bands are quite wide, which suggest that several vibrational modes contribute to these bands. This circumstance was taken into account when investigating the calculated and observed spectra. The simulated IR absorbance spectrum is shown in Figure 4a. Supposed assignment of the observed IR absorption bands to the calculated normal modes is presented in the Table 1.

The 3-deacetylcalonectrin molecule contains 46 atoms, resulting in 132 vibrational modes. In order to gain a better understanding of the spectral region of the most IR active modes, a set of the simple models with characteristic polar groups were tested. In the case of 3-deacetylcalonectrin, these models are ethyl acetate (see Figure 4), tetrahydropyran and cyclopentanol (see Figure S1). Vibrational modes with close frequencies form characteristic spectral features.

The highest-frequency peak of 3626 cm^−1^ in the experimental IR absorption spectra is attributed to the O–H bond stretching vibration. The calculated frequency of this mode is 3707 cm^−1^. Such overestimation of the frequency value is typically for these modes due to the high anharmonicity of the X–H bond stretching vibrations.

The experimental IR absorption spectrum exhibits a wide feature with a maximum at 2962 cm^−1^. Various C–H stretching vibrations are located in the range of 2800–3100 cm^−1^. The 2962 cm^−1^ absorption maximum corresponds to the antisymmetric vibrations of the methyl groups. The peak has well-pronounced shoulders at 2920 cm^−1^ and approximately 3030 cm^−1^. The former is related to the antisymmetric vibrations in the CH_2_ group. Interpretation of the latter requires further calculations. The additional shoulder near 2870 cm^−1^ corresponds to symmetric stretching vibrations in the CH_2_ and CH_3_ groups [32].

The corresponding vibrational modes in the calculated spectrum are located at 2949–3114 cm^−1^. The highest frequency mode at 3167 cm^−1^ corresponds to the antisymmetric ν(CH_2_) mode in the epoxy ring. Its frequency is predicted to be higher than the stretching vibration in ν(=C–H). The shoulder at 3030 cm^−1^ observed in the IR spectrum from [3] was interpreted as absorption associated with antisymmetric vibration in the epoxy group CH_2_ and the stretching vibration of hydrogen in the =C–H unit.

According to theory, the frequencies of the symmetric stretching vibrations in the CH_2_ and CH_3_ groups, as well as CH stretching vibrations, are 2949–3044 cm^−1^. For the vast majority of stretching hydrogen vibrational modes, the following relation (1) is predicted:

ν_as_(CH_2_) epoxy > ν(=C–H) > ν_as_(CH_3_), ν_as_(CH_2_) > ν_s_(CH_3_), ν_s_(CH_2_), ν(CH) (1)

**Figure 5:**
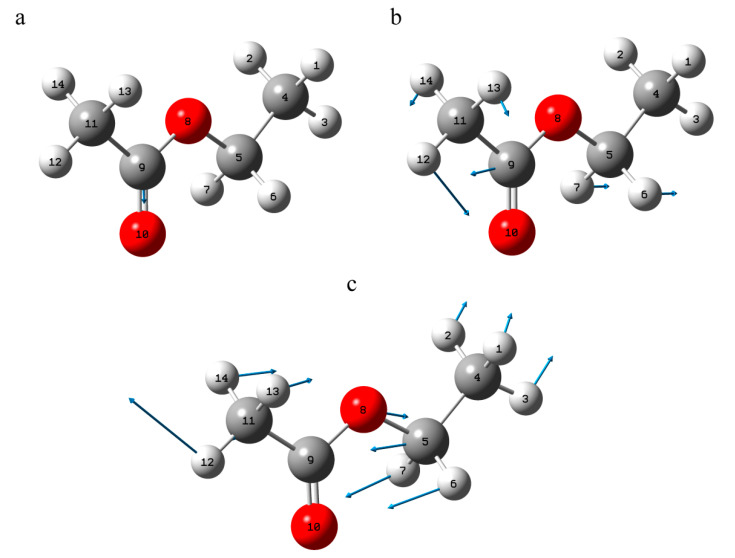
The most IR active vibrations in ethyl acetate molecule: 1772 (**a**), 1241 (**b**) and 1047 (**c**) cm^−1^.

The experimental IR spectrum of [3] contains an intense absorption peak at 1767 cm^−1^ and a weak satellite peak at 1709 cm^−1^, which were assigned to the v(C=O) and v(C=C), vibrations, respectively. The calculation predicts similar absorption spectrum with analogous peaks at 1772 cm^−1^ and 1703 cm^−1^. Calculations show that the vibration in the ethyl acetate molecule localized within the C=O bond has close frequency of 1772 cm^−1^ (see Figure 5).

The broad band near 1468 cm^−1^ in the experimental spectrum corresponds to three well-resolved bands, with maxima at 1495 cm^−1^, 1470 cm^−1^ and 1454 cm^−1^ in the calculated spectrum. These bands are related to the δ(CH_2_) modes and the asymmetric δ(CH_3_) modes. The coupling of such vibrational modes is due to the closeness of their frequencies, similar to situations in other organic compounds [32].

The absorption feature at 1387 cm^−1^ in the experimental spectrum is rather wide (approximately 100 cm^−1^). Ten modes contribute to the IR absorption within this frequency interval in the calculated spectrum. Their cumulative effect gives rise to a spectral band with two maxima located at 1371 cm^–1^ and 1430 cm^–1^.

The 1371 cm^–1^ mode involves predominantly symmetric δ(CH_3_) vibrations. This agrees with the reported characteristic frequency of 1375 ± 10 cm^−1^ for the δ_s_(CH_3_) modes [32].

Several modes contribute to the IR absorption band at 1430 cm^−1^. These modes have a mixed character, with the main contributions from δ(HCH) and symmetric δ_s_(CH_3_) vibrations. Furthermore, a small addition of δ(CCH), δ(HCO) and δ(COH) was noticed. Notice that a value of 1430 cm^−1^ lies between the characteristic frequencies of the δ(CH_2_) (1450 ± 10 cm^−1^) and δ_s_(CH_3_) (1375 ± 10 cm^−1^) modes [32]. In the low frequency shoulder of the theoretical spectrum (1320–1357 cm^−1^), a significant contribution arises from the δ(CCH) vibrations. In these modes, the w(HCH) and τ(HCH) vibrations, having lower vibration frequencies, also begin to participate.

The most intense band in the experimental absorbance spectrum is located at 1227 cm^−1^. The analogous band in the calculated IR spectrum corresponds to the vibrational mode, with a frequency of 1233 cm^−1^_._ This is the stretching vibration of the single C41-O40 bond in the acetate group. Due to the high polarity of the C–O bond, this mode is very active in IR absorption. More precisely, this is an asymmetric bond stretching mode localized within the C–C–O bridge. The mode also includes some contribution from the δ(H^44^C^43^C^41^) bending vibration, with the small addition of δ(H^45^C^43^H^46^) and w(H^30^C^29^H^31^) vibrations. The most IR-active mode of the ethyl acetate molecule has exactly the same atomic displacements and frequency. The difference is within 0.7%.

Near the intense IR peak, there is a weak band at 1177 cm^−1^. It can be assigned to the superposition of three lines around 1160 cm^−1^ in the calculated IR spectrum. These modes are delocalized over the whole molecule. A characteristic feature of these modes is the contribution of the C–C bond stretching vibrations of the four-fold coordinated C atoms.

Another significant band in the experimental absorption spectrum of 3-deactylcalonectrin is located at 1054 cm^−1^. The corresponding vibrational modes in the theoretical spectrum are located at 1013–1070 cm^−1^. Analysis of the calculated eigenvectors shows that the participating modes have two characteristic contributions (see Table 1). The first one is the hydrogen atom vibrations of a mostly rocking type, with the smaller addition of twisting and wagging types in the CH_2_ groups. Another contribution is due to non-hydrogen vibrations, especially localized in stretching single C–C and C–O bonds. The calculated vector of the dipole derivative is collinear with the C–O bonds.

It is predicted that the highest frequency vibrational mode in the region of 1013–1070 cm^−1^ is located at 1062 cm^−1^. The significant contribution to this mode comes from vibrations of the oxygen atom in the B-ring. The atomic displacements in the COC group are asymmetric and close to those observed for corresponding modes localized in the tetrahydropyran ring (see Figure S2 in Supporting Information).

According to calculations, there are two intense peaks in the IR spectrum of 3-deacetylcalonectrin in the region of 1013–1060 cm^−1^. Their frequencies are 1040 and 1044 cm^−1^. The 1040 cm^−1^ mode has a contribution of the v(C^29^O^40^) mode, localized in the acetate group. Similar atomic displacements were observed for a mode located at 1047 cm^−1^ in the ethyl acetate molecule. The greater difference, as compared with another modes in Table S2, can be explained by the significant atomic displacements that occur closer to the boundary carbon. This carbon atom, in the case of the 3-deacetylcalonectrin molecule, is terminal in R2, and through it the fused rings are attached.

The 1044 cm^−1^ mode has a significant contribution of the v(O16-C8) bond stretching vibration with the O atom within the hydroxyl group. Calculations predict a similar vibrational mode with a frequency of approximately 1076 cm^−1^ in the cyclopentanol (see Figure S3a).

Slightly weaker IR-active modes with frequencies of 1024, 1032 and 1061 cm^−1^ are predicted for the IR spectrum of 3-deacetylcalonectrin. They involve different stretching vibrations localized in the C–O bonds of the B-ring. A vibrational mode similar to the 1032 cm^−1^ mode is observed in the cyclopentanol model. It has atomic displacements localized in the C–O(H) bond and a close frequency of 1054 cm^−1^ (see Figure S3b).

The experimental absorption band at 974 cm^−1^ can be assigned to the mode with a frequency of 987 cm^−1^ in the calculated spectrum. In this mode, the atomic displacements occur mainly within the A-ring, with a characteristic v(C28C20) bond stretching vibration.

It is also worth noting that, in the calculated spectrum, an additional three modes are predicted with frequencies of 920, 955 and 962 cm^−1^. Their IR activity is comparable with that of the 987 cm^−1^ mode. The 962 cm^−1^ mode involves contributions of the v(C33O34), v(C18O34) and δ(C18O33C34) vibrations, localized in the epoxide ring. Both 920 and 955 cm^−1^ modes include contributions of the v(C–O) vibrations localized in the B-ring. The 920 cm^−1^ mode also contains significant contributions of the v(C41O40) vibration in the acetate group and the v(C29C19) and v(C34O33) vibrations in the epoxide group.

Modes in the region of 630–882 cm^−1^ include significant contributions of the v(C–C) and v(C–O) vibrations, delocalized over the fused rings accompanied by the rocking modes. These modes have low polarity and therefore low activity in the predicted IR spectrum.

Additional contributions from δ(CCC), δ(COC), δ(OCC) and δ(OCO) bending modes start to occur. These contributions become much more significant in the region of 630–300 cm^−1^, in which the ρ(HCH) contributions are also present.

Predominantly torsion modes are present below 300 cm^−1^. The most active ones in this region are those localized in the CCOH groups, i.e., of the tors(CCOH) type. The cyclopentanol model predicts that the τ(OH) torsional mode is located at 280 cm^−1^. This is close to the analogous vibrational mode (256 cm^−1^) in the calculated spectra of 3-deacetylcalonectrin. The relatively lower frequency of the mode in 3-deacetylcalonectrin can be explained by mixing with other torsional modes. Furthermore, some contributions of τ(OH) torsional vibrations can be found for the 370 and 388 cm^−1^ modes in the calculated spectrum of 3-deacetylcalonectrin.

#### 3.2.2. T-2 Toxin Molecule

As mentioned previously, the T-2 toxin molecule has several structural similarities with 3-deacetylcalonectrin and the model structures shown in Figure S1. The one common feature of the T-2 toxin is the presence of the 3-methylbutanoate group. In order to reveal a manifestation of structural similarities, it was decided to compare the predicted IR absorbance spectrum of the T-2 toxin with that predicted for methyl 3-methylbutanoate (see Figure S4). The predicted spectra for the T-2 toxin, 3-deacetylcalonectrin, ethyl acetate and methyl 3-methylbutanoate are compared in Figure 4.

Table 2 summarizes the most IR-intensive vibrational modes.

According to theory, the acetate group attached to the C-ring has a low influence on v(OH) vibration. The corresponding mode in the calculated scaled IR absorbance spectrum is located at 3710 cm^−1^. As in the case of 3-deacetylcalonectrin, the anharmonicity of the X–H bond stretching vibrations may also take place. Moreover, in [7], the short contact between the OH group of one molecule and the oxygen atom of another was highlighted.

For the T-2 toxin, various carbon–hydrogen stretching vibrations are located in the 2944–3116 cm^−1^ range, close to the similar region in 3-deacetylcalonectrin. For both substances, the frequencies of these modes are also overestimated due to neglect of anharmonicity.

The stretching C–H vibrational mode in the epoxy group possesses the highest frequency in this region. Its value is higher than in the case of the stretching vibrations of hydrogen bonded to sp^2^ carbon (v(=C–H)). As mentioned above, the relation (1) is valid for the majority of vibrational C-H stretching modes. However, the relation can be violated due to weak coupling of modes with the boundary frequency values. This takes place for the modes localized in the R1 and R3 groups of the T-2 toxin molecule. The predominantly asymmetric vibrations in CH_2_ and CH_3_ are located in the 3017–3170 cm^−1^ range.

**Figure 6:**
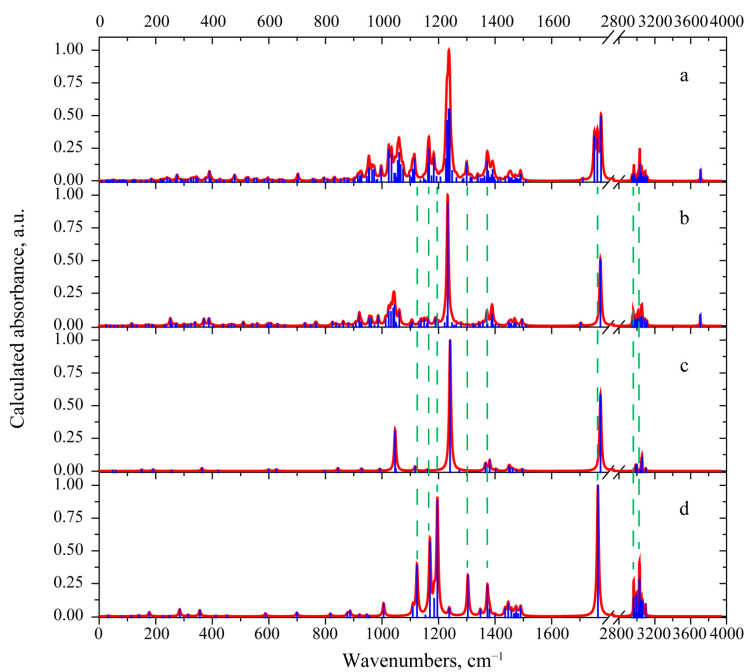
Calculated absorbance spectra for T-2 toxin (**a**), 3-deacetylcalonectrin (**b**), ethyl acetate (**c**) and methyl 3-methylbutanoate group (**d**).

In the 2944–3116 cm^−1^ range the main difference between the calculated IR spectra of the two types of toxin concerns the resulting band shape. In the case of the T-2 toxin, the maximal absorption is related to the vibrational modes localized in the 3-methylbutanoate group with antisymmetric stretching atomic displacements. These modes are located near 3029, 3033 and 3043 cm^−1^. Moreover, in the region of symmetric stretching vibrations among the three vibrational modes with the greatest absorption, two of them related the atomic displacements in the 3-methylbutanoate group, and the other one occurs due to symmetric vibrations in the CH_3_ group attached to the A-ring. The presence of similar highly active vibrational modes in the calculated IR absorbance spectrum of methyl 3-methylbutanoate leads to similar contour and maxima in that region (see Figure 6).

However, in the 2944–3116 cm^−1^ region, the contribution from other C–H bonds of the T-2 toxin should not be forgotten. In this region, the stretching vibrations v(CH) with carbon atoms situated at the A- and B-ring boundaries possess the lowest frequency at 2944 cm^−1^.

The theory predicts three intense IR absorption peaks near 1760 cm^−1^. These originate from the v(C=O) vibrations. Such vibration localized in the R2 group corresponds to the peak located at 1775 cm^−1^ and has the highest IR-intensity. A similar feature group is present in the spectrum of 3-deacetylcalonectrin close at 1772 cm^−1^. The v(C=O) vibration localized in the R3 group has a lower frequency and is located near to 1762 cm^−1^. The v(C=O) vibration with the lowest frequency (1751 cm^−1^) is localized in the 3-methylbutanoate group.

Analysis of the v(C=O) modes in the ethyl acetate and methyl 3-methylbutanoate model molecules provides frequencies of 1772 and 1764 cm^−1^, respectively. This is in qualitative agreement with the situation in the T-2 toxin molecule in which the v(C=O) mode localized in the R1 group has a lower frequency compared to those localized in the R2 and R3 groups.

Considering the T-2 toxin, the theory also predicts a double carbon stretching vibrational mode located at 1710 cm^−1^.

The modes with the main contribution of the δ(HCH) and δ_as_(CH_3_) deformational vibrations are located in the 1436–1509 cm^−1^ range. They give rise to a complex spectral feature, with three well-pronounced maxima at 1453, 1471 and 1490 cm^−1^. The modes which significantly contribute to IR-absorbance in this region are listed in Table 2.

The region of 1362–1418 cm^−1^ in the calculated IR absorption spectra exhibits two bands with maxima near 1372 and 1390 cm^−1^ (see Figure 6). The main contributions to the 1372 cm^−1^ band give two vibrational modes. The more IR-active one is located at 1372 cm^−1^. It is attributed to δ_s_(CH_3_) vibrations in the acetate groups. The second mode (less IR-active) is located near 1376 cm^−1^. It is assigned for various deformation vibrations with hydrogen atoms of δ(CCH) type. These δ(CCH) vibrations are distributed within the T-2 toxin molecule; several are present in the fused rings and other ones are present in the 3-methylbutanoate group. A similar vibration located at 1373 cm^−1^ can be found in the spectrum of the methyl 3-methylbutanoate molecule (see Table S3).

The 1390 cm^−1^ band in the spectrum of the T-2 toxin consists of two main contributions. The first of these is vibrational mode, with a frequency of 1391 cm^−1^. Its predominant atomic displacements are δ_s_(C^12^H_3_) and δ_s_(C^27^H_3_), and also δ(O^16^C^8^H^9^) and δ(C^8^O^16^H^17^). The second contribution is the vibrational mode located at 1387 cm^−1^. This is predominantly associated with δ(C^8^O^16^H^17^), δ(O^16^C^8^H^9^) and δ(O^18^C^10^H^11^) vibrations.

The theory predicts for the T-2 toxin, a characteristic IR-peak near 1300 cm^−1^, which is absent in the spectrum of 3-deacetylcalonectrin. It is related to the mode localized predominantly in the 3-methylbutanoate group and includes the w(H^51^C^49^H^50^), δ(C^53^C^52^H^55^), v(C^47^C^49^) and v(C^47^O^46^) vibrations. Comparison with the spectrum of the methyl 3-methylbutanoate model supports this hypothesis. In the calculated IR absorbance spectrum of the model molecule, there is a 1302 cm^−1^ mode which has a similar atomic pattern (see Table S3 and Figure 6).

The most intensive band in the theoretical IR absorbance spectrum of the T-2 toxin is located at 1237 cm^−1^. It is related to three modes with frequencies of 1239, 1236 and 1230 cm^−1^. Their characteristic feature is the prominent contribution of v(CO) in both acetate groups. In the case of the 1239 cm^−1^ vibrational mode, the predominant v(CO) occurs in the R2. A similar mode exists in the spectrum of 3-deacetylcalonectrin. Additionally, in this mode, τ(H^51^C^49^H^50^) atomic displacements take place. The 1230 cm^−1^ mode also has prominent v(CO) as well as (δ(C^40^C^42^H^45^) and δ(C^8^O^16^H^17^)), τ(H^50^C^49^H^51^) and tors(H^7^C^6^C^8^H^9^) contributions. In the case of 1236 cm^−1^, the predominant contribution is from v(CO) in the acetate groups of R3 as well as τ(H^50^C^49^H^51^), with a smaller addition of various bending deformational modes with hydrogen atoms. The calculated dipole derivative unit vector in such vibrational modes has a direction close to the CO bond in which predominant displacements occur. Hence, these modes were interpreted as predominantly v(CO) absorption in the R2 and R3 groups (not in fused rings and not in the 3-methylbutanoate group). This is supported by the presence of a highly IR active mode in the spectrum of ethyl acetate and at the same time the absence of IR active modes is in the same region in the spectrum of methyl 3-methylbutanoate (see Figure 6).

Characteristic spectral features for the T-2 toxin are the bands in the 1090–1200 cm^−1^ region. The common feature of intense IR absorbance modes in this region is the significant contribution of stretching vibrations in the 3-methylbutanoate group, which include stretching vibrations v(C^47^O^46^). The calculated frequencies of these modes are 1109, 1115, 1163, 1166 and 1183 cm^−1^. The 1183 cm^−1^ mode in the T-2 toxin has similar atomic displacements in the 3-methylbutanoate groups to the 1196 cm^−1^ mode in methyl 3-methylbutanoate.

Additionally, there are significant contributions for the 1109 cm^−1^ mode from stretching vibrations in the A-ring and τ(HCH) in the epoxy ring; for the 1163 cm^−1^ mode, there are w(HCH) in the epoxy ring and v(C^12^C^3^), and for the 1166 cm^−1^ mode there are w(HCH) in the epoxy ring as well as δ(HCC) and w(HCH) type vibrations in the 3-methylbutanoate group. The atomic displacements in 1166 and 1163 cm^−1^ modes in T-2 toxin are similar to 1170 cm^−1^ mode in methyl 3-mehtylbutanoate model. To summarize, presence of the IR-lines corresponding to the 1183, 1166 and 1163 cm^−1^ modes can be used as a spectroscopic fingerprint of the 3-methylbutanote group.

One can also discern a difference between the IR spectra of T-2 and 3-deacetylcalonectrin in the features at approximately 914 and 1075 cm^−1^. In these regions, in the T-2 toxin, there were vibrational modes where atomic displacements occurs predominantly in v(CO), v(CC) and ρ(HCH), as well as torsion modes including hydrogen. These modes can be classified on the basis of structures with oxygen in which the vibrations take place.

In the region of 1043–1075 cm^−1^, the calculation predicts a 1061 cm^−1^ mode, which has greater absorption. This is due to the v_as_(COC) vibrations within the B-ring oxygen. The same contribution was noted for the 1066 cm^−1^ mode. Moreover, some small contribution of the v(C^26^O^5^) vibrations localized in the B-ring was noted for the 955 and 953 cm^−1^ modes.

In the aforementioned region near the most IR-active mode, the modes with frequencies of 1075 and 1056 cm^−1^ are located. In these modes, stretching vibration occurs between carbon of the C-ring and oxygen of the hydroxyl group (v(C^8^O^16^)). In the case of the 1056 cm^−1^ mode, some additional contribution from stretching vibrations in the CO bonds of the epoxide ring was noticed. However, such contribution is more pronounced in the 965 cm^−1^ mode.

The highest absorbance in the 914–1043 cm^−1^ region is predicted for modes located at 1034, 1024, 997 and 972 cm^−1^. These modes have atomic displacements in groups that deal with oxygen in R1, R2 and R3.

Below 900 cm^−1^, the theory predicts the contribution of stretching vibrations v(CO) and v(CC) modes, mostly down to 615 cm^-1^. Considering the bending vibrations in groups CCC, CCC and CCC, most of them are located in the 730–300 cm^−1^ region. The modes in 615–730 cm^−1^ have a mixed stretching-bending character. The torsional modes predominantly contribute below 300 cm^−1^. However, a small contribution in mix with other vibrational types was noted from 1200 cm^−1^, in particular, in the case of that with hydrogen atoms (i.e., tors(ABCH), where A,B,C—some atoms). The contribution of ρ(HCH) continuously appears down to approximately 555 cm^−1^, being mixed with the stretching and bending vibrations of single bonds with carbon and oxygen.

In the region below 900 cm^−1^_,_ a set of vibrational modes located at 703, 478, 390, 275, 240 and 185 cm^−1^ is predicted. Comparing with others, these modes have the highest absorption, and from the experimental point of view and due to environmental effects, the most important of these for practical reasons are the modes with frequencies above 300 cm^−1^, namely the modes at 703, 478 and 390 cm^−1^. Their common feature is the contribution of vibration in groups with oxygen. The 703 cm^−1^ mode has a contribution of the δ(CCO) groups, the 479 cm^−1^ mode has a contribution of the δ(O^39^C^40^C^42^) and δ(O^5^C^26^C^1^) groups with a small addition of tors(C^6^C^8^O^16^H^17^) and the 390 cm^−1^ mode has a significant contribution of tors(C^6^C^8^O^16^H^17^).

### 3.3. UV-Vis Absorption with TD-DFT Approach

The absorption spectra in the UV-visible region of the T-2 toxin and 3-deacetylcalonectrin were simulated by the time-dependent density functional theory (TD-DFT) method using the exchange correlation potential B3LYP and the 6-31G(d,p) basis set. In both cases, the environment influence was taken into account implicitly by means of a polarizable continuum model with standard solvent properties. The choice of methanol as a solvent is explained by the studied sample solubility and the transparency in the considered spectral range, as well as a standardized technique for the absorption spectra registration. Furthermore, liquid chromatography experiments with methanol were tested in several different independent laboratories [33], which allows comparison of the obtained theoretical results with the experimental data presented in [1,33,34]. The singlet approach simulation of the 60 lowest excited states, as well as calculation of the oscillator strength for transitions between excited and ground state were performed. As a result, information on absorption for the T-2 toxin in the energy range from 2.6053 eV (475.90 nm) to 7.6618 eV (161.82 nm) and for 3-deacetylcalonectrin in the energy range from 2.4984 eV (496.25 nm) to 8.7945 eV (140.98 nm) was acquired.

**Figure 7:**
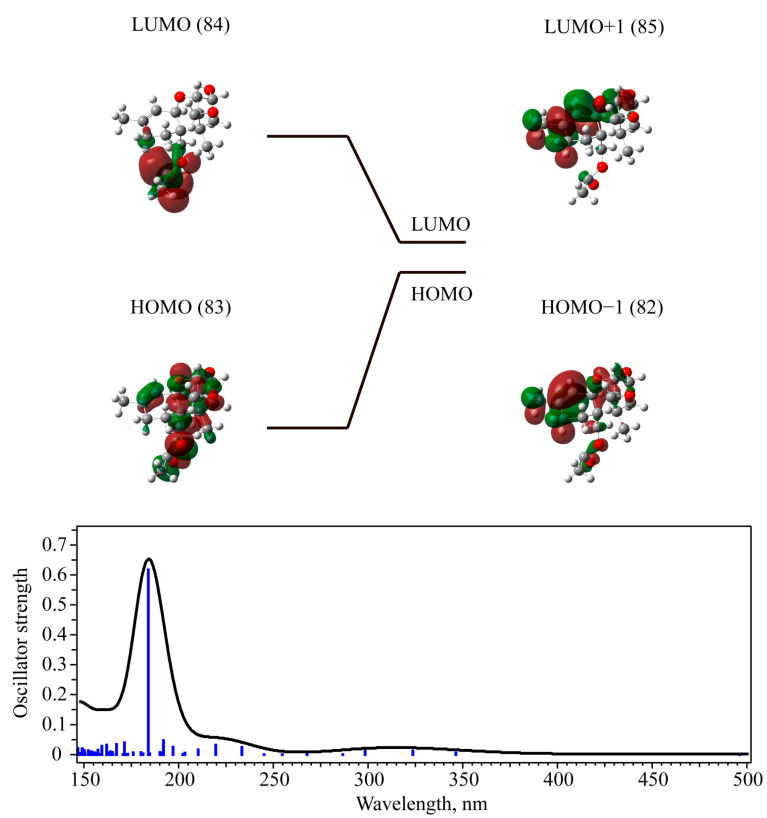
TD-DFT calculated molecular orbitals (HOMO-1 (82), HOMO (83), LUMO (84), LUMO+1 (85)) for 3-deacetylcalonectrin in methanol (**top**) and calculated UV-Vis absorbance spectra (**bottom**).

**Figure 8:**
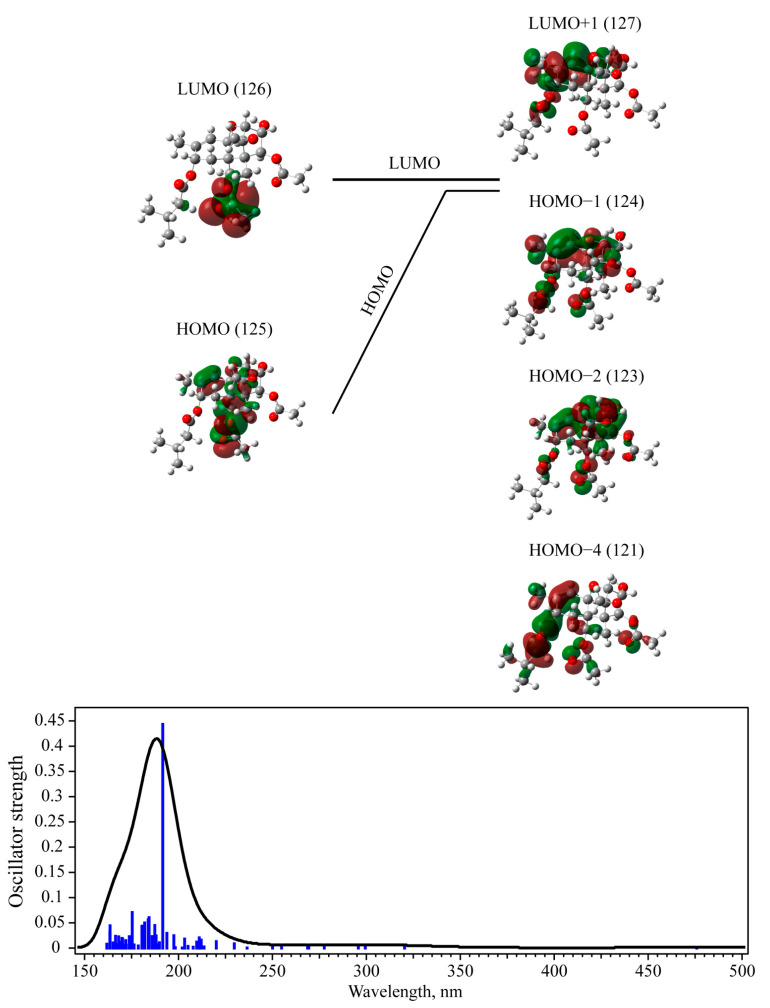
TD-DFT calculated selected molecular orbitals for T-2 toxin in methanol (**top**) and calculated UV-Vis absorbance spectra (**bottom**).

The first excited state for T-2 and 3-deacetylcalonectrin possesses the longest wavelength and corresponds to a linear combination of transitions between orbitals, with the maximal contribution from the transition between Highest Occupied Molecular Orbital (HOMO) and Lowest Unoccupied Molecular Orbital (LUMO) (see Figure 7 and Figure 8). This contribution is approximately 87% for the T-2 toxin and 75% for 3-deacetylcalonectrin. Nevertheless, the oscillator strength of this transition for both mycotoxins is extremely small and equals 0.0009 and 0.0005 for T-2 and 3-deacetylcalonectrin, respectively. It should be noted that, for most of the calculated transitions, the oscillator strength does not exceed 0.071 and 0.047 for T-2 and 3-deacetylcalonectrin, respectively.

The most intense 3-deacetylcalonectrin calculated transitions is 6.7390 eV (183.98 nm); its oscillator strength is 0.6168, which is more than an order of magnitude higher than for other transitions. The most pronounced contribution is related to the transition between orbitals 82 (HOMO−1) and 85 (LUMO+1) (Table 3). The highest change in the transition between these orbitals is associated with the electron density in the double carbon bond.

The π bonding character is noted for the 82 orbital, and the π* antibonding character is inherent to the 85 orbital. Thus, the discussed transition can be attributed as a π-π* transition. The 3-deacetylcalonectrin absorption spectrum is shown in Figure 7 (bottom).

The most intense transition among those calculated for the T-2 toxin is 6.4710 eV (191.60 nm); its oscillator strength, equal to 0.4432, is more than six times higher than the maximum oscillator strength for other transitions. In this case, the maximum contribution was achieved due to transitions from orbitals 124 (HOMO−1), 123 (HOMO−2) and 121 (HOMO−4) to orbital 127 (LUMO+1) (see Table 3 and Figure 8).

A characteristic similarity of the 124, 123 and 121 orbitals is the bonding nature of the electron density in the region of the C=C bond. In the 124 orbital, which has the greatest contribution to the transition, the most delocalized bonding region extends along the atoms from C=C in the A-ring, through C–O–C in the B-ring and ends at the carbon atom in the C-ring bound to the OH group. The bonding pattern at the double carbon bond corresponds to π bonding. During the transition to the 127 orbital at C=C, a distribution of electron density is noted similar to π* antibonding.

The transition with the highest oscillator strength in the T-2 toxin is similar to that in 3-deacetylcalonectrine in terms of the change in the C=C bond electron density. The main differences for the T-2 toxin are linked to a greater degree of delocalization within the trichothecene rings, as well as the presence of certain electron densities at the 3-methylbutanoate group, which results in a longer wavelength shift in absorption.

## 4. Conclusions

The molecular structure and vibrational spectra of 3-deacetylcalonectrin and the T-2 toxin were studied by computer modelling based on DFT calculation. Geometry optimization of 3-deacetylcalonectrin and the T-2 toxin provided stable configurations that were in good agreement in similar structural parts with experimental XRD data. It is determined that core units in both molecules have close structural parameters (bond length, angles). This supports the idea of the rigidity of fused rings. Several conformer configurations differing in orientations of the attached functional groups were found for the T-2 molecule.

The simulated IR spectrum of 3-deacetylcalonectrin is in good agreement with the experimental data. This result made it possible to assign all significant spectral feature to particular vibrational modes localized in different structural units. It was noticed that the most intense IR bands are associated with vibrations localized in the C–O and C=O bonds of the methyl acetate and acetate radicals. The modes with vibrations in the C–O and C=O bonds in the 3-methylbutanoate group have lower IR activity. Based on the computational results, it was possible to predict several spectral regions (approximately 1160–1185 cm^−1^ and 1300 cm^−1^) in which the characteristic vibrational modes of this group are expected.

The modelling of optical absorption spectra of 3-deacetylcalonectrin and T-2 toxins predicts only one significant π-π* transition associated with the electron density change localized in the C=C bond. The transition wavelength calculated for the T-2 toxin is close to the experimental value reported in several papers.

## Figures and Tables

**Table 1:** Selected experimental peaks and calculated modes for 3-deacetylcalonectrin. In the table, the following notations have been used: v—stretching vibrations, δ—bending vibrations, asym—asymmetric, sym—symmetric vibrations, w—wagging, tors—torsional, τ—twisting, ρ—rocking modes. In the case of water-like >CH_2_ structures, v_sym_ and v_asym_ correspond, respectively, to symmetric and asymmetric water-like stretching vibrations, with corresponding atoms in parentheses. The upper index denotes the atom number and lower index denotes the number of elements attached to the previous atom.

Mode No	Calculated Frequency, cm^−1^	Scaled Frequency, cm^−1^	IR Intensity, KM/Mole	Experiment [3]	Interpretation
15	256	252	24.8		predominantly tors(C^6^C^8^O^16^H^17^), tors(C^18^C^3^C^12^H^13^), tors(C^18^C^3^C^12^H^14^), tors(C^18^C^3^C^12^H^15^), tors(C^3^C^19^C^23^O^5^), tors(C^3^C^18^C^6^O^5^),
23	345	339	11.4		predominantly tors(C^6^C^8^O^16^H^17^), ρ(H^21^C^20^H^22^), ρ(H^31^C^29^H^30^), δ(C^1^C^2^C^24^), ρ(H^35^C^34^H^36^), δ(C^18^C^3^C^12^)
24	377	370	23.8		predominantly tors(C^6^C^8^O^16^H^17^) with small addition of δ(C^24^C^2^C^28^), ρ(H^32^C^28^H^38^), ρ(H^11^C^10^H^39^), δ(C^2^C^28^C^20^), δ(C^1^C^23^C^19^), δ(C^19^C^3^C^18^), δ(C^23^O^5^C^6^)
25	395	388	25.2		predominantly tors(C^6^C^8^O^16^H^17^), δ(C^43^C^41^O^40^), δ(C^24^C^2^C^28^), δ(C^20^C^19^C^3^), δ(C^19^C^3^O^10^)
39	740	728	9.2		δ(C^1^C^23^C^19^), δ(C^23^O^5^C^6^), ρ(H^32^C^28^H^38^), ρ(H^21^C^20^H^22^), ρ(H^31^C^29^H^30^), ρ(H^22^C^20^H^21^), tors(H^4^C^1^C^2^C^24^), v(C^18^C^3^), v(C^18^C^6^), ρ(H^36^C^34^H^35^), v(C^2^C^24^)
41	778	765	9.1		v(C^20^C^19^), v(C^19^C^3^), v(C^19^C^29^), v(C^2^C^28^), v(C^23^C^19^), ρ(H^38^C^28^H^32^), ρ(H^21^C^20^H^22^), ρ(H^11^C^10^H^39^), tors(H^4^C^1^C^2^C^24^)
43	839	826	13.1		v(C^6^O^5^), v(C^23^O^5^), v(C^18^O^33^), v(C^2^C^28^), v(C^2^C^24^), tors(H^4^C^1^C^2^C^28^), ρ(H^39^C^10^H^11^)
46	878	863	15.3		v(C^18^O^33^), v(C^18^C^6^), v(C^34^O^33^), ρ(H^13^C^12^H^14^), ρ(H^15^C^12^H^14^), τ(H^35^C^34^H^36^), ρ(H^31^C^29^H^30^), tors(H^4^C^1^C^2^C^28^)
49	923	908	10.5		ρ(H^14^C^12^H^13^), ρ(H^14^C^12^H^15^), ρ(H^27^C^24^H^26^), ρ(H^27^C^24^H^25^), ρ(H^32^C^28^H^38^), ρ(H^11^C^10^H^39^), w(H^22^C^20^H^21^), v(O^40^C^41^)
50	936	920	41.4		v(C^41^O^40^), v(C^23^O^5^), v(C^34^O^33^), v(C^29^C^19^), v(C^41^C^43^), ρ(H^39^C^10^H^11^), ρ(H^32^C^28^H^38^), ρ(H^22^C^20^H^21^), τ (H^35^C^34^H^36^), tors(C^19^C^23^C^1^H^4^)
51	942	926	11.9		τ (H^35^C^34^H^36^), v(C^41^O^40^), v(C^2^C^28^), v(C^20^C^28^), ρ(H^27^C^24^H^25^), ρ(H^27^C^24^H^26^), v(C^6^C^18^)
53	972	955	27.5		v(C^23^O^5^), ρ(H^32^C^28^H^38^), τ (H^22^C^20^H^21^), τ (H^27^C^24^H^25^), v(C^23^C^1^), v(C^6^O^5^), ρ(H^13^C^12^H^15^), ρ(H^14^C^12^H^15^)
54	979	962	24.8		v(C^33^O^34^), v(C^18^O^34^), δ(C^18^O^33^C^34^), ρ(H^35^C^34^H^36^), v(C^10^C^8^), δ(H^7^C^6^C^18^), ρ(H^14^C^12^H^15^), ρ(H^14^C^12^H^13^)
56	1004	987	33.1	974	v(C^28^C^20^), v(C^23^C^19^), δ(C^2^C^1^C^23^), w(H^32^C^28^H^38^), ρ(H^21^C^20^H^22^), with small addition of v(O^40^C^41^), ρ(H^26^C^24^H^27^), ρ(H^25^C^24^H^27^), ρ(H^15^C^12^H^14^), ρ(H^13^C^12^H^14^)
57	1031	1013	23.1	1013	δ(H^21^C^20^C^28^), ρ(H^25^C^24^H^27^), ρ(H^26^C^24^H^25^), w(H^32^C^28^H^38^), ρ(H^15^C^12^H^14^), ρ(H^15^C^12^H^13^) ρ(H^10^C^11^H^39^), v(C^19^C^23^), v(C^10^C^8^), v(C^20^C^28^)
58	1041	1024	51.3		v(C^28^C^20^), v(C^29^O^40^), v(C^1^C^23^), ρ(H^27^C^24^H^25^), ρ(H^27^C^24^H^26^), v(C^23^O^5^), v(C^6^O^5^)
59	1050	1032	49.6		v(C^23^O^5^), v(O^5^C^6^), v(C^19^C^23^), v(C^8^C^10^), v(C^3^C^12^), v(C^1^C^23^), ρ(H^25^C^24^H^27^), ρ(H^26^C^24^H^25^), ρ(H^15^C^12^H^14^), ρ(H^15^C^12^H^13^)
60	1058	1040	61.3		predominantly v(C^29^O^40^), ρ(H^44^C^43^H^46^), ρ(H^44^C^43^H^45^), δ(C^43^C^41^O^40^), τ(H^32^C^28^H^38^), ρ(H^26^C^24^H^27^), ρ(H^26^C^24^H^25^), τ(H^11^C^10^H^39^), δ(C^32^C^28^H^20^), w(H^22^C^20^H^21^), tors(C^24^C^2^C^1^H^4^)
61	1062	1044	72.9		v(O^16^C^8^), v(C^8^C^10^), τ(H^11^C^10^H^39^), δ(H^17^O^16^C^8^), ρ(H^26^C^24^H^25^), ρ(H^27^C^24^H^26^), ρ(H^30^C^29^H^31^), δ(C^8^C^16^C^18^),
62	1067	1049	11.7		predominantly ρ(H^26^C^24^H^25^), ρ(H^27^C^24^H^26^), ρ(H^45^C^43^H^44^), ρ(H^45^C^43^H^46^), tors(C^43^C^41^O^40^C^29^), v(C^8^O^16^) τ (H^11^C^10^H^39^)
64	1080	1061	47.9		v(C^6^O^5^), v(C^23^O^5^), v(C^6^C^8^), δ(H^17^O^16^C^8^), δ(H^11^C^10^C^3^), ρ(H^13^C^12^H^14^), ρ(H^13^C^12^H^15^), ρ(H^35^C^34^H^36^)
67	1126	1107	11.6		ρ(H^31^C^29^H^30^), ρ(H^22^C^20^H^21^), ρ(H^32^C^28^H^38^), τ(H^36^C^34^H^35^), δ(C^29^C^19^C^20^), v(C^3^C^19^), τ(H^25^C^24^H^26^)
69	1158	1138	19.9		v(C^6^C^18^), v(C^3^C^12^), w(H^36^C^34^H^35^), δ(H^39^C^10^C^8^), δ(H^9^C^8^C^10^), δ(H^17^O^16^C^8^), v(C^8^O^16^), τ (H^21^C^20^H^22^)
70	1169	1149	21.8		v(C^3^C^10^), v(C^3^C^19^), ρ(H^15^C^12^H^13^), ρ(H^15^C^12^H^14^), δ(H^37^C^23^C^1^), δ(H^4^C^1^C^23^), τ (H^32^C^28^H^38^), τ (H^11^C^10^H^39^), δ(H^27^C^24^C^2^), w(H^36^C^34^H^35^)
72	1182	1162	20.3		v(C^24^C^2^), δ(H^4^C^1^C^23^), δ(C^23^C^1^C^2^), w(H^36^C^34^H^35^), δ(H^11^C^10^C^8^), δ(H^9^C^8^C^6^), w(H^32^C^28^H^38^), δ(H^17^O^16^C^8^)
73	1209	1189	24.0	1173	v(C^20^C^19^), v(C^23^C^19^), v(C^30^C^10^), τ (H^31^C^29^H^30^) δ(H^11^C^10^C^3^), ρ(H^15^C^12^H^13^), ρ(H^15^C^12^H^14^), δ(H^32^C^28^C^2^)
74	1219	1198	10.5		tors(H^9^C^8^C^10^H^39^), δ(H^17^O^6^C^8^), δ(H^9^C^8^C^6^), δ(H^7^C^6^C^18^) with small addition of v(C^20^C^19^), v(C^19^C^3^), v(C^19^C^23^)
76	1251	1230	32.7		tors(H^7^C^6^O^5^C^23^), δ(H^17^O^16^C^8^), δ(H^9^C^8^O^16^), w(H^36^C^34^H^35^), τ (H^21^C^20^H^22^), v(C^18^C^34^), δ(H^17^C^28^C^20^)
77	1254	1233	437	1227	predominantly v(O^40^C^41^), δ(H^44^C^43^C^41^) with small addition of δ(H^45^C^43^H^46^), w (H^30^C^29^H^31^)
87	1380	1357	11.1	1387	δ(H^37^C^23^O^5^), w(H^22^C^20^H^21^), δ(H^17^O^16^C^8^), δ(H^7^C^6^C^8^)
88	1395	1371	45.6		predominantly δ_sym_(C^43^H_3_)
90	1411	1387	26.3		predominantly δ(C^19^C^23^H^37^), δ(H^17^O^16^C^8^), δ(H^9^C^8^O^16^), δ(C^23^C^1^H^4^), δ(C^28^C^20^H^21^), w(H^32^C^28^H^38^) w(H^30^C^29^H^31^),
91	1414	1390	40.4		δ_sym_(C^12^H_3_), w(H^30^C^29^H^31^), δ_sym_(C^24^H_3_), δ(C^19^C^23^H^37^), δ(H^17^O^16^C^8^), δ(H^9^C^8^O^16^) δ(H^35^C^34^C^36^)
96	1474	1449	11.0	1468	δ_asym_(C^43^H_3_),
101	1493	1468	10.6		δ_asym_(C^24^H_3_), δ(H^32^C^28^H^38^), δ_asym_(C^12^H_3_)
102	1495	1470	11.6		δ(H^11^C^10^H^39^), δ(H^21^C^20^H^22^), δ(H^32^C^28^H^38^)
105	1521	1495	21.6		δ(H^30^C^29^H^31^), δ_asym_(C^12^H_3_)
107	1732	1703	10.5	1709	v(C^1^=C^2^)
108	1803	1772	236.3	1767	v(C^41^=O^42^)
109	3000	2949	15.1	3626	v(C^23^H^37^)
110	3003	2952	16.6		v_sym_(H^38^C^28^H^32^) small addition of v_sym_(C^24^H_3_), v(C^23^H^37^)
111	3011	2960	46.5		v_sym_(C^24^H_3_), small addition of v_sym_(H^38^C^28^H^32^)
113	3044	2992	16. 8		v(C^8^H^9^) small addition v(C^10^H^39^)
114	3047	2995	14.4		v_sym_(C^12^H_3_)
117	3065	3013	25.0		v_sym_(H^11^C^10^H^39^) small addition of v_sym_(H^21^C^20^H^22^), v_sym_(H^31^C^29^H^30^), v(C^8^H^9^)
118	3066	3014	24.5		v_sym_(H^21^C^20^H^22^)
120	3083	3031	20.6		v_sym_(H^35^C^34^H^36^)
121	3096	3044	29.2		v(C^6^H^7^)
122	3101	3048	17.6		v_asym_(C^24^H_3_)
123	3103	3050	22.3		v_asym_(H^22^C^20^H^21^), v_asym_(C^12^H_3_), v_asym_(H^11^C^10^H^39^)
125	3107	3054	26.2		v_asym_(H^11^C^10^H^39^), v_asym_(H^31^C^29^H^30^), v_asym_(H^22^C^20^H^21^)
126	3111	3058	32.6		v_asym_(H^13^C^12^H^15^), v_asym_(H^21^C^20^H^22^), v_asym_(H^31^C^29^H^30^)
128	3116	3063	20.6		v_asym_(C^12^H_3_), v_asym_(H^31^C^29^H^30^), v_asym_(H^11^C^10^H^39^)
129	3141	3088	22.0		v(=C^1^-H^4^)
131	3167	3114	15.6		v_asym_(H^35^C^34^H^36^)
132	3771	3707	38.3	3626	v(OH)

**Table 2:** Assignment of selected vibrational modes of the T-2 toxin molecule for calculated IR absorbance spectra. In the table, the following notations have been used: v—stretching vibrations, δ—bending vibrations, w—wagging, tors—torsional, τ—twisting, ρ—rocking modes, asym—asymmetric, sym—symmetric vibrations. In the case of water-like >CH_2_ structures, v_sym_ and v_asym_ correspond to symmetric and asymmetric water-like stretching vibrations, with corresponding atoms in parentheses. The upper index denotes the atom number and lower index denotes the number of elements attached to the previous atom.

Mode No	Calculated Frequency, cm^−1^	Scaled Frequency, cm^−1^	IR Intensity, KM/Mole	Interpretation
42	396	390	34.7	tors(C^6^C^8^O^16^H^17^), δ(C^26^O^5^C^6^), δ(C^22^C^3^O^21^)
49	487	479	23.0	δ(O^39^C^40^C^42^), δ(C^27^C^2^C^1^), δ(O^5^C^26^C^1^), tors(C^6^C^8^O^16^H^17^), v(C^10^C^8^), v(C^39^C^36^)
63	715	703	26.3	δ(C^12^C^3^C^10^), v(C^21^C^3^), δ(C^3^C^10^O^18^), δ(C^6^C^8^O^16^), ρ(H^25^C^23^H^24^), v(C^10^C^3^), v(C^12^C^3^), v(C^21^C^3^), δ(C^8^C^6^O^5^), v(C^64^C^21^), v(C^6^C^21^)
82	969	953	49.5	v(C^26^O^5^), ρ(H^59^C^54^H^61^), ρ(H^60^C^54^H^61^), ρ(H^57^C^53^H^58^), ρ(H^57^C^53^H^56^), δ(C^53^C^52^H^55^), v(C^16^C^26^), ρ(H^30^C^27^H^29^), ρ(H^30^C^27^H^28^)
83	971	955	43.5	ρ(H^59^C^54^H^61^), ρ(H^60^C^54^H^61^), ρ(H^57^C^53^H^58^), ρ(H^57^C^53^H^56^), v(C^52^C^53^), v(C^26^O^5^), v(C^6^C^8^), tors (C^31^C^2^C^1^H^4^)
84	982	965	40.9	v(C^64^O^63^), v(C^21^O^63^), δ(C^64^O^63^C^21^), ρ(H^56^C^53^H^57^), v(C^10^C^8^), tors(C^64^C^21^C^6^H^7^)
85	989	972	41.4	v(C^47^O^46^), v(C^47^-C^49^H_2_), ρ(H^56^C^53^H^57^), δ(H^59^C^54^C^52^), ρ(H^30^C^27^H^29^), ρ(H^30^C^27^H^28^), ρ(H^25^C^23^H^24^)
88	1015	997	51.7	v(C^26^C^22^), v(O^5^C^26^), v(C^47^O^46^), ρ(H^28^C^27^H^30^), ρ(H^28^C^27^H^29^), ρ(H^25^C^23^H^24^), ρ(H^37^C^36^H^38^), δ(C^26^C^22^C^23^)
89	1042	1024	120.0	v(C^31^O^46^), v(C^10^O^18^), v(C^1^C^26^), v(C^3^C^12^), v(C^22^C^26^), ρ(H^35^C^32^H^33^), ρ(H^35^C^32^H^34^), ρ(H^37^C^36^H^38^), ρ(H^24^C^23^H^25^), ρ(H^44^C^42^H^45^)
90	1052	1034	99.5	v(C^10^O^18^), ρ(H^33^C^32^H^35^), ρ(H^34^C^32^H^35^), τ(H^44^C^42^H^45^), ρ(H^24^C^23^H^25^), ρ(H^13^C^12^H^15^), ρ(H^14^C^12^H^15^), ρ(H^30^C^27^H^29^), ρ(H^30^C^27^H^28^), v(C^22^C^26^), δ(H^4^C^1^C^2^)
95	1074	1056	76.0	v(C^8^O^16^), v(C^8^C^10^), v(C^64^O^63^), v(C^21^O^63^), ρ(H^33^C^32^H^34^), ρ(H^35^C^32^H^34^), δ(H^7^C^6^C^21^), δ(C^8^C^6^C^21^)
96	1079	1061	106.4	v(C^6^O^5^), v(C^26^O^5^), v(C^36^O^39^), v(C^23^C^31^), v(C^8^O^16^), ρ(H^44^C^42^H^45^), ρ(H^43^C^42^H^45^), ρ(H^65^C^64^H^66^), tors (C^21^C^6^C^8^C^10^), δ(H^17^O^16^C^8^)
97	1085	1066	60.8	v(C^10^O^18^), v(C^31^C^23^), v(C^6^O^5^), v(C^26^O^5^), ρ(H^28^C^27^H^30^), ρ(H^29^C^27^H^30^), tors(C^31^C^2^C^1^H^4^), tors(C^31^C^2^C^27^H^28^), tors(H^67^C^26^C^1^H^4^)
98	1093	1075	43.6	v(C^8^O^16^), ρ(H^15^C^12^H^13^), ρ(H^14^C^12^H^13^), v(C^8^C^10^), v(C^8^C^6^), δ(H^11^C^10^C^3^), ρ(H^37^C^36^H^38^), ρ(H^24^C^25^H^23^)
102	1128	1109	42.5	v(C^23^C^22^), v(C^46^C^47^), v(C^1^C^26^), v(C^2^C^27^), v(C^47^O^46^), τ (H^65^C^64^H^66^), tors(H^4^C^1^C^26^H^67^), δ(H^50^C^49^C^47^), ρ(H^24^C^23^H^25^), ρ(H^15^C^12^H^13^), tors(H^59^C^54^C^52^H^55^), δ(H^58^C^53^C^52^)
103	1134	1115	82.3	v(C^47^O^46^), tors(H^50^C^49^C^52^H^55^), δ(H^58^C^53^C^52^), δ(H^59^C^54^C^52^), τ(H^57^C^53^H^58^), w(H^61^C^54^H^60^)
107	1183	1163	66.4	v(C^47^O^46^), v(C^52^C^49^), v(C^12^C^3^), w(H^66^C^64^H^65^), w(H^51^C^49^H^50^), ρ(H^57^C^53^H^56^), ρ(H^57^C^53^H^58^), ρ(H^59^C^54^H^61^), ρ(H^59^C^54^H^60^), tors(H^55^C^52^C^53^H^57^), tors(H^55^C^52^C^54^H^61^), δ(H^9^C^8^C^6^), δ(H^11^C^10^C^8^), δ(H^7^C^6^C^21^), δ(H^61^C^54^C^52^)
108	1187	1166	122.8	v(C^47^O^46^), w(H^51^C^49^H^50^), δ(H^55^C^52^C^49^), δ(H^57^C^53^C^52^), δ(H^61^C^54^C^52^), w(H^58^C^53^H^56^), τ(H^60^C^54^H^59^), v(C^52^C^49^), w(H^66^C^64^H^65^), tors(H^9^C^8^C^10^H^11^)
110	1204	1183	87.7	v(C^47^O^46^), δ(C^53^C^52^C^54^), w(H^60^C^54^H^61^), w(H^57^C^53^H^58^), δ(H^56^C^53^C^52^), δ(H^59^C^54^C^52^), δ(H^55^C^52^C^49^), δ(H^50^C^49^C^52^), δ(H^51^C^49^C^47^), w(H^51^C^49^H^50^)
114	1251	1230	229.3	v(C^39^O^40^), δ(C^40^C^42^H^45^) with addition of tors(H^7^C^6^C^8^H^9^), δ(C^8^O^16^H^17^), τ(H^50^C^49^H^51^)
115	1257	1236	273.1	predominantly v(C^19^O^18^), τ(H^50^C^49^H^51^) with addition of δ(C^19^C^32^H^35^), δ(C^6^C^8^H^9^), δ(C^21^C^6^H^7^), δ(C^8^O^16^H^17^), δ(C^54^C^52^H^55^)
116	1261	1239	273.4	predominantly v(C^40^O^39^), v(C^19^O^18^), v(C^47^O^46^), τ(H^51^C^49^H^50^) with addition of tors(H^55^C^52^C^54^H^60^)
121	1322	1300	68.4	predominantly w(H^51^C^49^H^50^), δ(C^53^C^52^H^55^), v(C^47^C^49^), v(C^47^O^46^) with small addition of δ(C^2^C^31^H^62^)
130	1395	1372	74.6	predominantly δ_sym_ (C^42^H_3_), δ_sym_ (C^32^H_3_)
131	1400	1376	35.9	δ(C^22^C^26^H^67^), δ(C^2^C^31^H^62^), δ(C^22^C^26^H^67^), δ(C^49^C^52^H^55^), δ(C^52^C^49^H^50^), δ(C^26^C^1^H^4^) with small addition of δ_sym_ (C^27^H_3_), δ_sym_ (C^32^H_3_)
134	1411	1387	21.8	δ(C^8^O^16^H^17^), δ(O^16^C^8^H^9^), δ(O^18^C^10^H^11^) with small addition of δ_sym_ (C^32^H_3_)
135	1415	1391	41.3	δ_sym_ (C^12^H_3_), δ_sym_ (C^27^H_3_), δ(O^16^C^8^H^9^), δ(C^8^O^16^H^17^)
143	1473	1448	16.3	δ_asym_ (C^32^H_3_)
147	1481	1456	10.6	δ_asym_(C^42^H_3_) with small addition of δ(H^25^C^23^H^24^), δ_asym_ (C^27^H_3_)
150	1496	1471	12.1	δ_asym_(C^27^H_3_) with small addition of δ(C^2^C^1^H^4^)
156	1516	1491	24.7	δ(H^37^C^36^H^38^), δ_asym_(C^12^H_3_)
158	1740	1710	8.0	predominantly v(C^1^=C^2^) with small addition of δ(H^4^C^1^C^2^)
159	1781	1751	170.1	v(C^47^=O^48^)
160	1792	1762	162.1	v(C^19^=O^20^)
161	1805	1775	245.4	v(C^40^=O^41^)
162	2995	2944	18.3	v(C^26^H^67^)
163	3015	2964	25.3	anti-phase v_sym_(C^54^H_3_) v_sym_(C^53^H_3_)
164	3018	2966	23.9	in-phase v_sym_(C^54^H_3_), v_sym_(C^53^H_3_), v(C^52^H^55^) with small addition of v_sym_(H^51^C^49^H^50^)
165	3020	2969	24.8	v_sym_(C^27^H_3_)
177	3081	3029	41.9	v_asym_(C^53^H_3_), v_asym_(H^51^C^49^H^50^), v_asym_(H^61^C^54^H^60^), v(C^52^H^55^)
180	3085	3033	63.6	v_asym_(C^54^H_3_), v_asym_(C^53^H_3_), v(C^52^H^55^), v_asym_(H^50^C^49^H^51^)
181	3096	3043	32.9	v_asym_(C^54^H_3_)
194	3170	3116	13.8	v_asym_(H^65^C^64^H^66^)
195	3774	3710	42.1	v(OH)

**Table 3:** Comparison of theoretical and experimental results for UV absorbance spectra.

Compound	Transition Corresponding to Maximum Oscillator Strength				
	Type	Oscillator Strength	Orbitals with Contribution >5% (%)	Wavelength, nm (Energy, eV)	Experimental Value from [1,33]
T-2 toxin	Singlet	0.4432	124 -> 127 (54%) 123 -> 127 (19%) 121 -> 127 (9%)	192 (6.471)	203 (6.109)
3-deacetylcalonectrin	Singlet	0.6168	82 -> 85 (88%)	184 (6.739)	

## Supplementary Materials

The following are available online at https://www.mdpi.com/article/10.3390/ma15020649/s1, Figure S1: Optimized structures of several models: ethyl acetate (a), tetrahydropyran (b), cyclopentanol (c), Figure S2: The tetrahydropyran mode with significant contribution of asymmetric C8O1C5 stretching vibrations, Figure S3: Selected vibrational modes calculated for cyclopentanol models structure: 1076 (a), 1054 (b) and 280 (c) cm^−1^, Figure S4: Optimized structure of methyl 3-methylbutanoate, Table S1: Selected structural parameters of T-2 and 3-deacetylcalonectrin molecules. The notations are taken from Figure 2 and Figure 3, Table S2: The comparison of selected calculated modes for model structures: ethyl acetate, tetrahydropyran, cyclopentanol with scaled calculated frequency for 3-deacetylcalonectrin, Table S3: The comparison of selected calculated modes for methyl 3-methylbutanoate with scaled calculated frequency for T-2 toxin.
