# Supplementary material for: DFT Modelling of Molecular Structure, Vibrational and UV-Vis Absorption Spectra of T-2 Toxin and 3-Deacetylcalonectrin
Source: Materials (Basel). 2022 Jan 15;15(2):649. doi: 10.3390/ma15020649 (PMC8780109; doi:10.3390/ma15020649)
Supplement: Supplementary file 1 [file materials-15-00649-s001.zip › materials-1494960-supplementary.pdf]

Supporting information for

DFT modelling of T-2 toxin and 3-deacetylcalonectrin structures and their vibrational and UV-Vis absorption spectra

Table S1. Selected structural parameters of T-2 and 3-deacetylcalonectrin molecules. The notations are taken from figure 2 and figure 3

		**T-2 toxin**					**3-deacetylcalonectrin**	
		**Theory**			**Experiment**			**Theory**
		**Notations**	**Conformer 1**	**Conformer 2**	**Molecule 1**	**Molecule 2**	**Notations**	**Lowest found conformer**
A-ring	Bonds, Å	C1-C2	1.330	1.330	1.320	1.317	C1-C2	1.331
		C2-C31	1.505	1.507	1.493	1.469	C2-C28	1.507
		C31-C23	1.530	1.529	1.510	1.509	C28-C20	1.532
		C23-C22	1.544	1.544	1.549	1.552	C20-C19	1.545
		C22-C26	1.556	1.556	1.547	1.549	C19-C23	1.555
		C26-C1	1.501	1.501	1.481	1.493	C23-C1	1.503
	Angles, °	C1-C2-C31	120.9	120.8	121.4	121.7	C1-C2-C28	121.1
		C2-C31-C23	114.5	114.4	113.9	115.2	C2-C28-C20	113.1
		C31-C23-C22	115.0	115.1	114.4	115.6	C28-C20-C19	112.5
		C23-C22-C26	108.0	107.8	107.9	107.0	C20-C19-C23	107.6
		C22-C26-C1	114.4	114.4	114.2	114.4	C19-C23-C1	114.1
		C26-C1-C2	125.3	125.4	125.4	124.8	C23-C1-C2	124.9
	Dihedral angles, °	C1-C2-C31-C23	9.3	9.1	12.9	8.6	C1-C2-C28-C20	15.7
		C2-C31-C23-C22	-38.7	-38.7	-41.2	-36.6	C2-C28-C20-C19	-46.6
		C31-C23-C22-C26	54.0	54.2	54.9	52.5	C28-C20-C19-C23	59.2
		C23-C22-C26-C1	-41.9	-42.2	-42.6	-42.6	C20-C19-C23-C1	-42.2
		C22-C26-C1-C2	16.3	16.5	16.9	19.2	C19-C23-C1-C2	14.1
		C26-C1-C2-C31	1.6	1.7	-1.0	-0.1	C23-C1-C2-C28	0.5
B-ring	Bonds, Å	O5-C26	1.453	1.453	1.444	1.451	O5-C23	1.454
		C22-C3	1.593	1.593	1.589	1.580	C19-C3	1.585
		C3-C21	1.535	1.534	1.526	1.527	C3-C18	1.533
		C21-C6	1.505	1.506	1.485	1.487	C18-C6	1.508
		C6-O5	1.428	1.428	1.422	1.418	C6-O5	1.429
	Angles, °	O5-C26-C22	114.2	114.1	113.6	112.0	O5-C23-C19	113.6
		C26-C22-C3	108.6	108.6	108.7	110.1	C23-C19-C3	108.7
		C22-C3-C21	107.3	107.3	106.4	106.0	C19-C3-C18	107.1
		C3-C21-C6	103.6	103.6	103.4	103.8	C3-C18-C6	103.4
		C21-C6-O5	110.8	110.9	110.1	109.8	C18-C6-O5	110.7
		C6-O5-C26	116.3	116.3	115.3	114.8	C6-O5-C23	116.4
	Dihedral angles, °	O5-C26-C22-C3	-42.9	-43.1	-44.0	-45.5	O5-C23-C19-C3	-43.6
		C26-C22-C3-C21	57.2	57.4	57.1	57.2	C23-C19-C3-C18	58.3
		C22-C3-C21-C6	-69.0	-69.0	-69.7	-68.1	C19-C3-C18-C6	-69.3
		C3-C21-C6-O5	68.6	68.4	71.1	71.1	C3-C18-C6-O5	68.4
		C21-C6-O5-C26	-56.8	-56.8	-60.3	61.6	C18-C6-O5-C23	-57.0
		C6-O5-C26-C22	43.5	43.6	45.9	47.6	C6-O5-C23-C19	44.0
	Bonds, Å	C8-C6	1.537	1.537	1.518	1.528	C8-C6	1.542
		C3-C10	1.578	1.579	1.563	1.563	C3-C10	1.568
		C10-C8	1.551	1.551	1.522	1.529	C10-C8	1.557

C-ring	Angles, ^{O}	C6-C21-C3	103.6	103.6	103.4	103.8	C6-C18-C3	103.4
		C21-C3-C10	99.8	99.8	100.6	100.1	C18-C3-C10	99.0
		C3-C10-C8	106.6	106.6	105.8	105.9	C3-C10-C8	107.2
		C10-C8-C6	104.2	104.2	105.4	105.6	C10-C8-C6	103.7
		C8-C6-C21	101.3	101.3	101.1	100.8	C8-C6-C18	101.5
	Dihedral angles, ^{o}	C6-C21-C3-C10	44.0	44.0	42.8	44.5	C6-C18-C3-C10	46.2
		C21-C3-C10-C8	-21.6	-21.5	-20.2	-22. 8	C18-C3-C10-C8	-25.4
		C3-C10-C8-C6	-7.4	-7.6	-8.6	-5.8	C3-C10-C8-C6	-3.4
		C10-C8-C6-C21	34.3	34.5	35. 0	32.9	C10-C8-C6-C18	31.7
		C8-C6-C21-C3	-49.9	-50.0	-48.9	-48.8	C8-C6-C18-C3	-50.0
E-ring	Bonds, Å	C21-O63	1.436	1.436	1.453	1.454	C18-O33	1.438
		O63-C64	1.437	1.437	1.444	1.462	O33-C34	1.438
		C21-C64	1.460	1.460	1.460	1.448	C34-C18	1.459
	Angles, ^{O}	C64-C21-C3	128.6	128.6	128.1	127.7	C34-C18-C3	128.6
	Dihedral angles, ^{o}	C64-C21-C3-C10	-156.0	-156.0	-155.5	-154.9	C34-C18-C3-C10	-153.3
R1	Bonds, Å	C31-O46	1.462	1.462	1.470	1.479		
		O46-C47	1.353	1.356	1.346	1.328		
		C47-O48	1.206	1.205	1.202	1.215		
		O48-H62	2.295	2.195				
	Angles, ^{O}	C54-C52-C53	111.2	111.2	104.1	109.5		
	Dihedral angles, ^{o}	C23-C31-O46-C47	87.3	119.1	83.4	118.0		
		C31-O46-C47-C49	179.4	-177.5	172.0	-175.7		
		O46-C47-C49-C52	-154.466	-154.419	-77.705	-87.2		
		C47-C49-C52-C53	-168.6	-168.6	-180.3	-174.1		
R2	Bonds, Å	C22-C36	1.542	1.543	1.532	1.546	C19-C29	1.541
		C36-O39	1.443	1.442	1.442	1.458	C29-O40	1.446
		C40-C39	1.353	1.352	1.338	1.331	O40-C41	1.353
		C40-C41	1.202	1.203	1.175	1.187	C41-O42	1.203
		O41-H37	2.708	2.872			O42-H30	2.585
		O41-H38	2.479	2.385			O42-H31	2.596
	Dihedral angles, ^{o}	C3-C22-C36-O39	71.5	72.1	73.2	72.8	C3-C19-C29-O40	79.6
		C22-C36-O39-C40	172.6	161.8	169.9	169.0	C19-C29-O40-C41	179.9
		C36-O39-C40-C42	173. 9	176.2	178.6	180.5	C29-O40-C41-C43	179.8
R3	Bonds, Å	C10-O18	1.442	1.442	1.439	1.460		
		C19-O18	1.354	1.354	1.355	1.337		
		C19-O20	1.205	1.205	1.177	1.206		
		O20-H11	2.289	2.281				
	Dihedral angles, ^{o}	C8-C10-O18-C19	-143.5	-142.4	-138.8	-116.7		
R4	Bonds, Å	C8-O16	1.415	1.415	1.410	1.422	C8-O16	1.420
		O5-H17	2.221	2.221			O5-H17	2.189
	Angles, ^{O}	C6-C8-O16	113.3	113.3	112.4	116.7	C6-C8-O16	112.7
	Dihedral angles, ^{o}	H17-O16-C8-C6	-10.2	-10.3			H17-O16-C8-C6	-5.4

Table S2. The comparison of selected calculated modes for model structures: ethyl acetate, tetrahydropyran, cyclopentanol with scaled calculated frequency for 3-deacetylcalonectrin.

**Model**	**Calculated frequency for model structure, cm^{-1}**	**Calculated scaled frequency for model structure, cm^{-1}**	**3-deacetylcalonectrin similar vibrational mode unscaled frequency, cm^{-1}**	**Type of significant contribution in model (see notations at figure S1)**
Ethyl acetate	1065	1047	1040	v(C5O8),
	1262	1241	1233	
	1803	1772	1772	
Tetrahydropyran	1101	1082	1061	asym(C8O1C5)
Cyclopentanol	285	280	252, 370, 388	tors(C4C3O15H16)
	1072	1054	1032	v(C3C2)
	1095	1076	1044	v(C3O15)

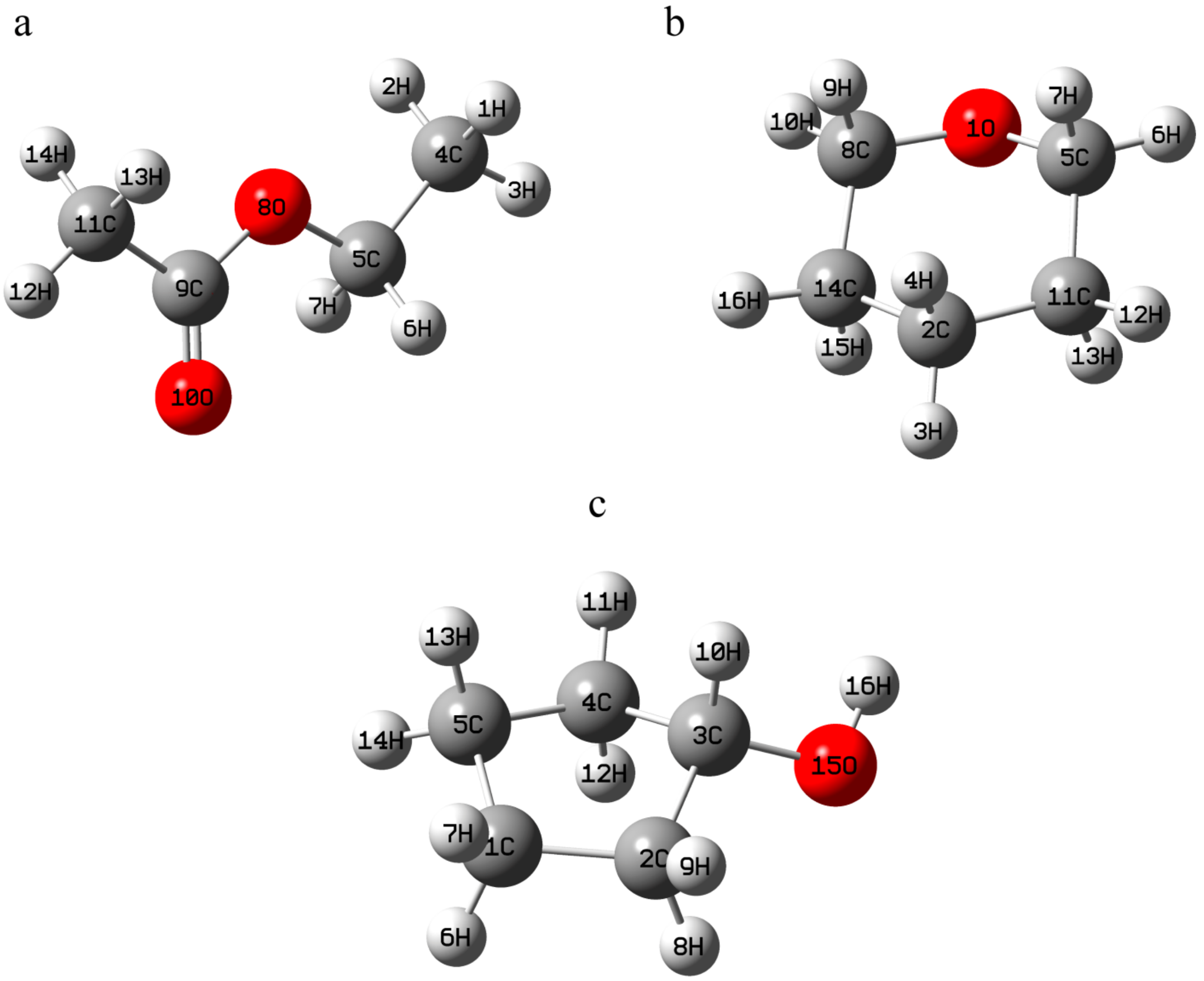

Figure S1. Optimized structures of several models: ethyl acetate (a), tetrahydropyran (b), cyclopentanol (c).

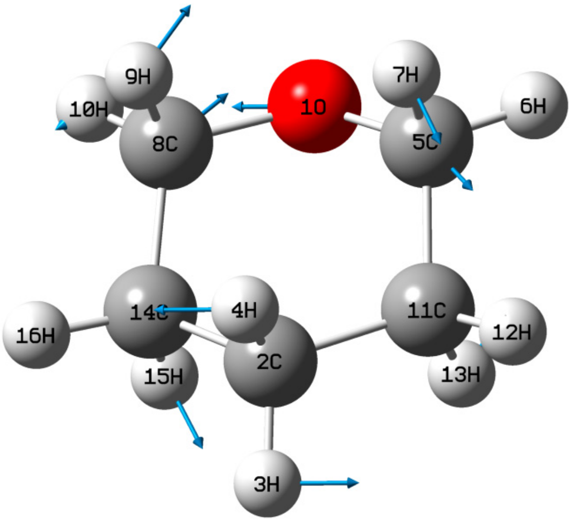

Figure S2. The tetrahydropyran mode with significant contribution of asymmetric C8O1C5 stretching vibrations.

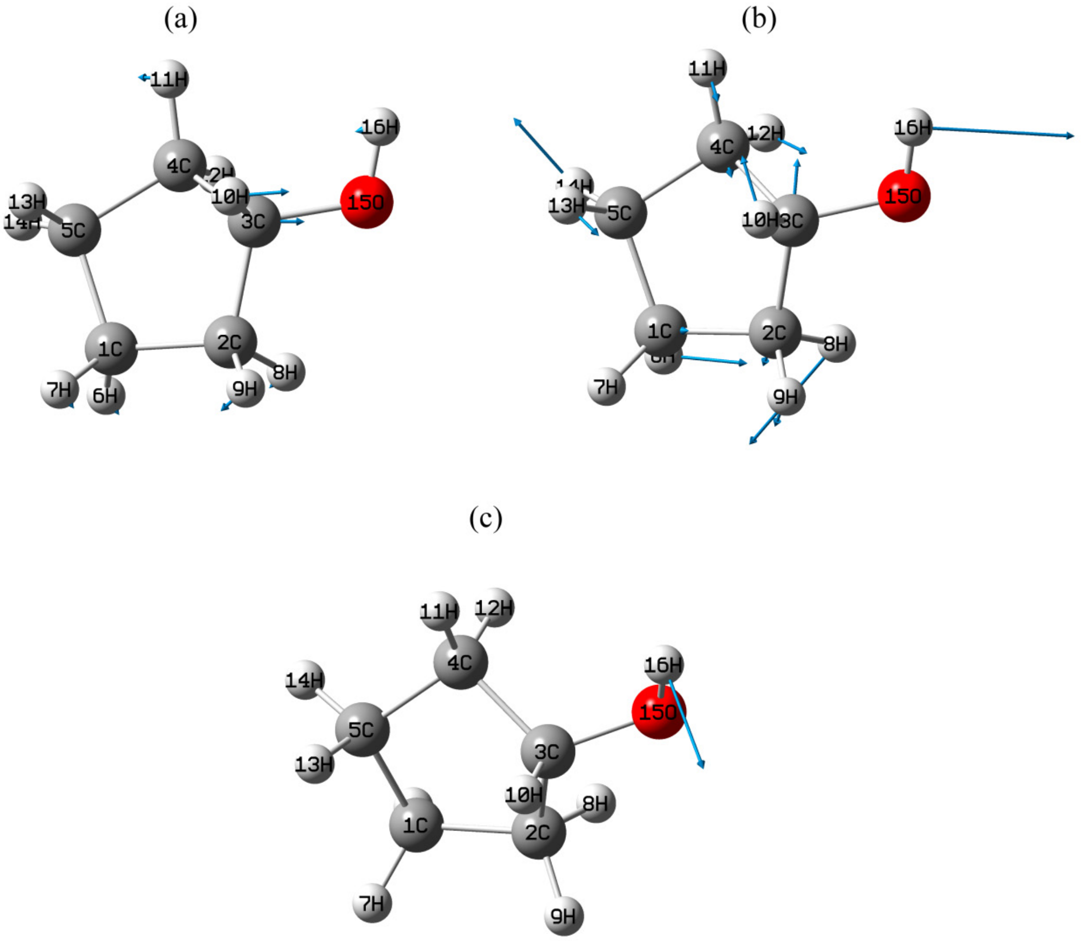

Figure S3. Selected vibrational modes calculated for cyclopentanol models structure: 1076(**a**),1054(**b**) and 280 (**c**) cm-1

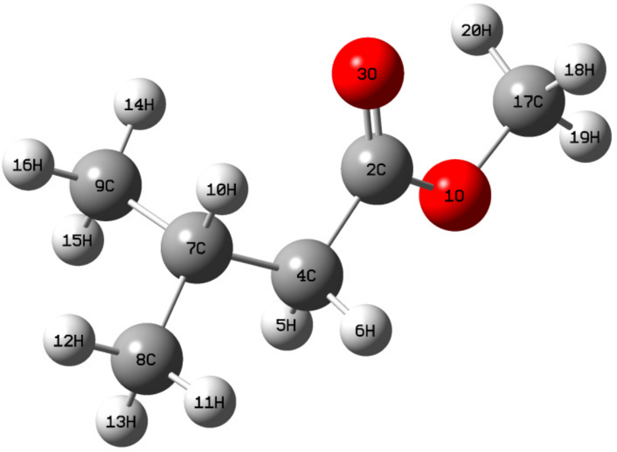

Figure S4. Optimized structure of methyl 3-methylbutanoate.

Table S3. The comparison of selected calculated modes for methyl 3-methylbutanoate with scaled calculated frequency for T-2 toxin.

Model	Calculated frequency for model structure, cm^{-1}	Calculated scaled frequency for model structure, cm^{-1}	T-2 toxin similar vibrational mode unscaled frequency, cm^{-1}	Type of significant contribution in model (see notations at figure S4)
Methyl 3-methylbutanoate	1143	1124	1109	predominantly $\nu(C^2O^1)$, $\delta(H^7C^8C^{13})$, $\delta(H^{10}C^7C^9)$, $\delta(H^5C^4C^2)$
	1190	1170	1166<br>1163	$\nu(C^2O^1)$, $\nu(C^7C^4)$, $w(H^5C^4H^6)$, $\delta(H^{10}C^7C^4)$, $\varrho(H^{12}C^8H^{11})$, $\varrho(H^{12}C^8H^{13})$, $\varrho(H^{14}C^9H^{16})$, $\varrho(H^{14}C^9H^{15})$
	1217	1196	1183	$\nu(C^2O^1)$, $w(H^{20}C^{17}H^{18})$, $w(H^5C^4H^6)$, $\delta(H^{10}C^7C^8)$, $\delta(H^5C^4C^7)$, $\delta(H^{13}C^8C^7)$, $\delta(C^9C^7C^8)$
	1326	1303	1300	predominantly $w(H^5C^4H^6)$, $\delta(C^8C^7H^{10})$, $\nu(C^4C^2)$,$\nu(C^2O^1)$ with addition of $\varrho(H^{13}C^8H^{11})$, $\varrho(H^{13}C^8H^{12})$
	1397	1373	1376	$\delta(H^{10}C^7C^4)$ $\delta(H^5C^4C^7)$
	1794	1764		$\nu(CO)$
	3013	2962	2964	anti-phase $\nu_{sym}(C^8H_3)$ $\nu_{sym}(C^9H_3)$
	3016	2965	2966	in-phase $\nu_{sym}(C^8H_3)$, $\nu_{sym}(C^9H_3)$, $\nu(C^7H^{10})$ with small addition of $\nu_{sym}(H^5C^4H^6)$
	3079	3027	3029	$\nu_{asym}(C^8H_3)$, $\nu(H^{19}C^9H^{15})$, $\nu(H^5C^4H^6)$, $\nu(C^7H^{10})$
	3084	3032	3033	$\nu_{asym}(C^8H_3)$, $\nu_{asym}(C^9H_3)$, $\nu(C^7H^{10})$
